# Serum Proteomic Analysis of Cannabis Use Disorder in Male Patients

**DOI:** 10.3390/molecules26175311

**Published:** 2021-09-01

**Authors:** Fawaz Alasmari, Sary Alsanea, Assim A. Alfadda, Ibrahim O. Alanazi, Mohthash Musambil, Afshan Masood, Faleh Alqahtani, Omer I. Fantoukh, Abdullah F. Alasmari, Hicham Benabdelkamel

**Affiliations:** 1 Department of Pharmacology and Toxicology, College of Pharmacy, King Saud University, Riyadh 11451, Saudi Arabia; ffalasmari@ksu.edu.sa (F.A.); salsanea@ksu.edu.sa (S.A.); afaleh@ksu.edu.sa (F.A.); afalasmari@ksu.edu.sa (A.F.A.); 2 Proteomics Resource Unit, Obesity Research Center, College of Medicine, King Saud University, Riyadh 11461, Saudi Arabia; aalfadda@ksu.edu.sa (A.A.A.); mthammitone@ksu.edu.sa (M.M.); afsmasood@ksu.edu.sa (A.M.); 3 Department of Medicine, College of Medicine, King Saud University, Riyadh 11461, Saudi Arabia; 4 National Center for Biotechnology (NCB), Life Science and Environment Research Institute, King Abdulaziz City for Science and Technology (KACST), Riyadh 11442, Saudi Arabia; ialenazi@kacst.edu.sa; 5 Department of Pharmacognosy, College of Pharmacy, King Saud University, Riyadh 11451, Saudi Arabia; ofantoukh@ksu.edu.sa

**Keywords:** serum proteomes, cannabis use disorder, proteomic profiling, LXR/RXR activation, FXR/RXR activation, acute phase responses, inflammation, atherosclerosis signaling

## Abstract

Cannabis use has been growing recently and it is legally consumed in many countries. Cannabis has a variety of phytochemicals including cannabinoids, which might impair the peripheral systems responses affecting inflammatory and immunological pathways. However, the exact signaling pathways that induce these effects need further understanding. The objective of this study is to investigate the serum proteomic profiling in patients diagnosed with cannabis use disorder (CUD) as compared with healthy control subjects. The novelty of our study is to highlight the differentially changes proteins in the serum of CUD patients. Certain proteins can be targeted in the future to attenuate the toxicological effects of cannabis. Blood samples were collected from 20 male individuals: 10 healthy controls and 10 CUD patients. An untargeted proteomic technique employing two-dimensional difference in gel electrophoresis coupled with mass spectrometry was employed in this study to assess the differentially expressed proteins. The proteomic analysis identified a total of 121 proteins that showed significant changes in protein expression between CUD patients (experimental group) and healthy individuals (control group). For instance, the serum expression of inactive tyrosine protein kinase PEAK1 and tumor necrosis factor alpha-induced protein 3 were increased in CUD group. In contrast, the serum expression of transthyretin and serotransferrin were reduced in CUD group. Among these proteins, 55 proteins were significantly upregulated and 66 proteins significantly downregulated in CUD patients as compared with healthy control group. Ingenuity pathway analysis (IPA) found that these differentially expressed proteins are linked to p38MAPK, interleukin 12 complex, nuclear factor-κB, and other signaling pathways. Our work indicates that the differentially expressed serum proteins between CUD and control groups are correlated to liver X receptor/retinoid X receptor (RXR), farnesoid X receptor/RXR activation, and acute phase response signaling.

## 1. Introduction

*Cannabis sativa* L. and *Cannabis indica* L. contain a variety of secondary metabolites. Cannabis plants species differ based on many factors, including the quantity of cannabinoids. Some of them are psychoactive and induce hallucinating effects such as delta-9-tetrahydrocannabinol (THC) while the others are non-psychoactive such as cannabidiol (CBD) [1]. The complexity of cannabis makes its use censorious because cannabis users may develop unexpected side effects, including central nervous system (CNS) side effects due to certain chemical ingredients. Therefore, exposure to cannabis means that the users will expose to several cannabinoids (~60) that are associated with pharmacological effects. In addition, the duration of exposure is a critical factor that significantly affects the quantity of these cannabinoids in the body.

Despite the presence of multiple compounds, cannabinoids family are the most abundant phytochemicals present in the cannabis plants [1]. Psychoactive effects resulted from cannabis exposure have been linked to THC [2]. This compound can modulate the processing of visual and auditory hallucination effects [3]. However, regulatory agencies have approved few cannabinoids to be used for certain indications. For instance, the U.S. Food and Drug Administration (FDA) has approved products containing CBD for seizures associated with Dravet syndrome and Lennox–Gastaut syndrome in one-year-old and older patients reported previously in clinical studies [4,5]. Moreover, FDA-approved synthetic products containing THC for the treatment of vomiting and nausea caused by chemotherapy treatments in patients who lack the response to conventional antiemetic treatments [6]. In addition, they can be prescribed to manage anorexia-associated with weight loss in patients diagnosed with acquired immunodeficiency syndrome [7].

Cannabis use disorder (CUD) is widespread across numerous countries [8]. The hallucination effects of cannabis use leads to drug abuse [9]. Governments and regulatory agencies set guidelines and policies to minimize the undesirable effects of cannabis [10,11]. As some countries have legalized the use of marijuana, smoking products containing marijuana are legally marketed nowadays.

Studies have documented toxicological effects in different models exposed to cannabis ingredients [12,13,14,15,16]. A recent study reported that high-grade atrioventricular block was developed in a young male following chronic exposure to marijuana [14]. Moreover, a recent case series study concluded that vaping cannabis oil was associated with acute respiratory depression [15]. It is important to consider that tachycardia and neurotoxicity were reported after acute inhalation of cannabis in humans [16]. Fivefold increase in blood carboxyhemoglobin levels were found in subjects who smoked marijuana for at least five years as compared with those who smoke tobacco cigarettes [13]. This study also noted that the burden of tar and carbon monoxide in the respiratory system has increased in marijuana smokers as compared with those who smoked a similar quantity of tobacco. On the other hand, CBD showed the ability to regulate immunological responses using in vivo and in vitro assays [17]. Moreover, CBD exhibits antioxidant and anti-inflammatory properties [18,19,20]. These anti-inflammatory effects were also found in non-psychoactive cannabinoids [20].

Several reports have determined the serum proteomic profiling of humans exposed to amphetamine analogs [21,22]. A recent study from our group identified differentially expressed proteins in the serum of individuals with amphetamine use disorder compared with a healthy control group [23]. Moreover, prior clinical proteomic studies utilized serum samples to determine the levels of proteins in patients who had developed neurodegenerative diseases [24,25], neurodevelopmental disorders [26,27,28], major depressive disorder, and bipolar disorders [29,30]. In the present study, we investigate the expression changes of CUD patients’ serum proteins compared with healthy controls, using an untargeted proteomic approach employing two-dimensional (2D) alteration in gel electrophoresis (2D-DIGE) coupled with mass spectroscopy (MS).

## 2. Results

### 2.1. Demographic Information

Demographic and clinical information of all participants included in our study has been collected. This information includes marital and employment status, age, gender, history of cannabis use disorder, and route of cannabis administration (Table 1).

### 2.2. Identification of Differentially Expressed Proteins and 2D-DIGE Analysis

**Figure 1:**
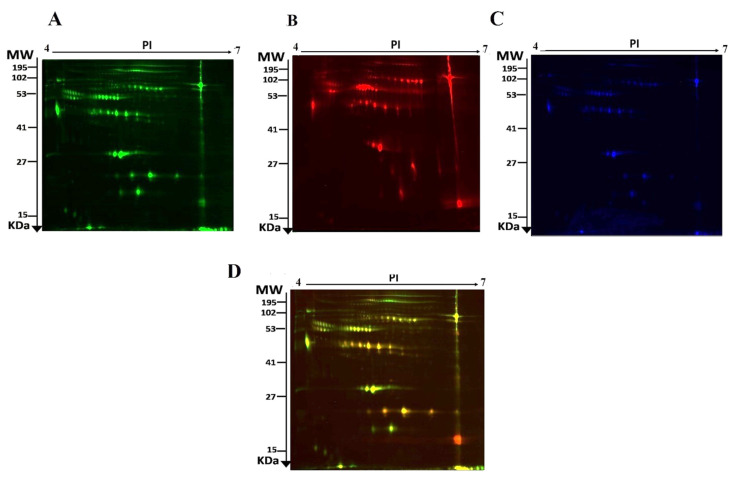
Representative fluorescent proteins of a two-dimensional difference in gel electrophoresis (2D-DIGE) containing serum sample from Control labeled with Cy3 (**A**), Cannabis labeled with Cy5 (**B**), Pooled internal control labeled with Cy2 (**C**), and merged image (**D**).

**Figure 2:**
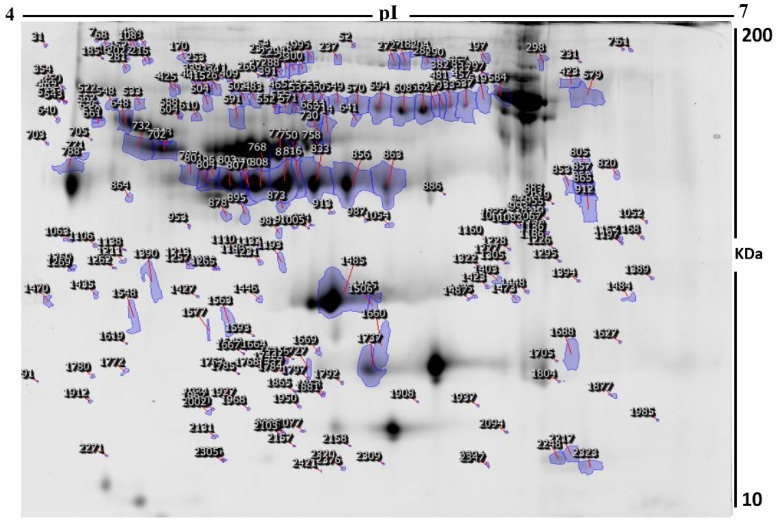
Fluorescence labeled (CyDyes)-2D-DIGE numbered spots indicate those proteins that were identified to be differentially abundant (defined as fold-change >1.5, *p* < 0.05) between the two groups (controls and CUD). These were successfully identified with matrix-assisted laser desorption/ionization time of flight (MALDI-TOF) mass spectrometry (MS). MW, protein molecular weight; pI, isoelectric point.

The current study assessed the difference in protein expression among 10 cannabis-exposed individuals and 10 controls (20 samples from 10 gels) using 2D-DIGE analysis technique before statistical analysis is performed with Progenesis software. Fluorescent protein profiles of a 2D-DIGE of control samples labelled with Cy3 are presented in Figure 1A. The CUD samples were labeled with Cy5 (Figure 1B), pooled internal control labeled with Cy2 (Figure 1C), and overlap of 2D-DIGE gels of samples labeled with Cy3/Cy5 (Figure 1D). A total of 1700 spots were identified on the gels, 156 were significantly different (ANOVA, *p* ≤ 0.05; fold-change ≥ 1.5) between the CUD and control groups (Figure 2). For alignment and further analysis, the spot patterns were reproducible across all 10 gels. The internal standard Cy2-labeled was included to perform normalization among the whole gels set in addition to the quantitative of the protein levels differential analysis. A total of 156 spots displayed a statistical significance among the two groups. These spots were manually excised from the preparative gel and underwent protein identification using MS.

**Figure 3:**
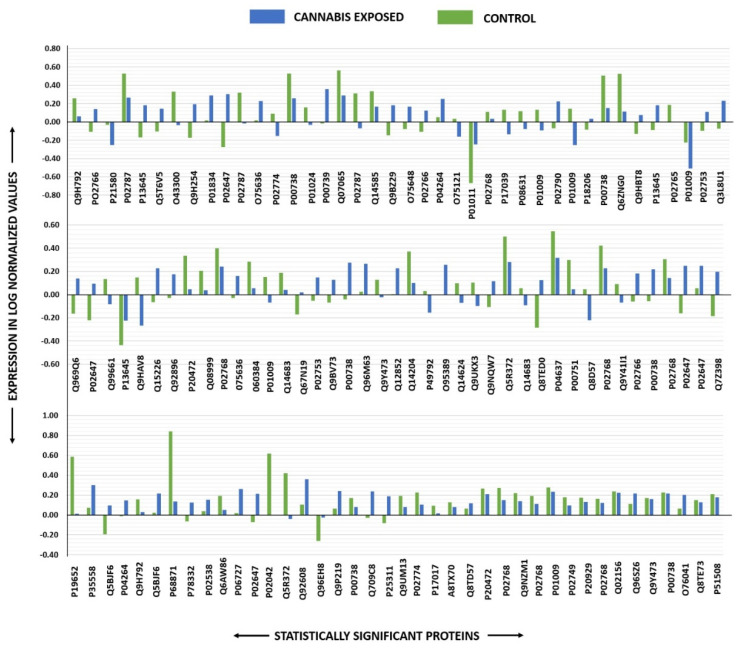
Differential expression of statistically significant protein spots from controls and cannabis-exposed samples.

Peptide mass fingerprints (PMFs) identified 121 out of 156 protein spots that were excised from preparative gel, MALDI-TOF. MS found 85 spots to be unique protein sequences. These sequences were matched to the SWISS-PROT database by Mascot search engine with high confidence scores (Table 2, Supplementary Table S2). The sequence coverage ranged from 4% to 85%. In few cases, the same protein variants were found at several locations on the gel (Table 2, Figure 2). Among the 121 proteins identified, 55 were upregulated and 66 were downregulated in the samples of CUD patients compared with that in the control subjects (Table 2, Figure 3). The significantly upregulated proteins included Apolipoprotein A-I (up 3.8-fold, *p* = 0.01), Alpha-1-antichymotrypsin (up 2.7-fold, *p* = 0.02), U3 small nucleolar RNA-associated protein 15 homolog (up 2.6-fold, *p* = 0.03), Zinc finger protein 550 (up 2.4-fold, *p* = 0.04), Haptoglobin-related protein (up 2.4-fold, *p* = 0.01), Spectrin beta chain, non-erythrocytic 4 (up 2.3-fold, *p* = 0.01), Keratin, type I cytoskeletal 10 (up 2.2-fold, *p* = 0.007), Dedicator of cytokinesis protein 9 (up 2.1-fold, *p* = 0.02), Haptoglobin (up 2.4-fold, *p* = 0.01), and Serine/threonine-protein phosphatase 2A regulatory subunit B’’ subunit gamma (up 2.0 fold, *p* = 0.05); a complete list is provided in Table 2. By contrast, the significantly downregulated proteins in CUD subjects included Hemoglobin subunit beta (down 5.0-fold, *p* = 0.05), Alpha-1-acid glycoprotein 2 (down 3.7-fold, *p* = 0.04), Rab GTPase-activating protein 1-like (down 2.9-fold, *p* = 0.02), and Ubiquitin domain-containing protein 1 (down 2.6-fold, *p* = 0.04) (Table 2, Supplementary Table S2). Among identified proteins: Inactive tyrosine protein kinase PEAK1, Transthyretin; Serotransferrin; Keratin, type I cytoskeletal 10; Apolipoprotein A-I; Ficolin-3; Vitamin D-binding protein; Haptoglobin; Keratin, type II cytoskeletal 1; Albumin; Alpha-1-antitrypsin; Retinol-binding protein 4, Outer dense fiber protein 2; Dynein heavy chain 3, axonemal; Parvalbumin alpha; Rab GTPase-activating protein 1-like; Structural maintenance of chromosomes protein 1A and Zinc finger protein 175 were found in ≥1 spot on the gels, which could be explained by post-translational modifications, cleavage by enzymes, or different protein species presence.

### 2.3. Principal Component Analysis

**Figure 4:**
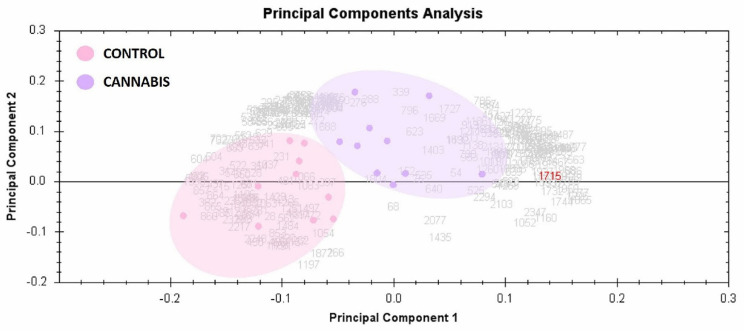
Principal component analysis of the proteomic dataset. Principal component analysis is presented in the figure where purple dots are the plasma samples from cannabis group and pink dots are from control group. Together, these explained 64% of the selected spot’s variability values. Colored dots and numbers are represented of the gels and spots, respectively.

To determine and visualize the CUD and control subjects’ samples, the principal component analysis of the Progenesis SameSpots software was used. The analysis was made on all 121 spots that exhibited statistically significant changes in abundance identified by MS. The analysis shows that the two groups clustered distinctly based on different proteins with score of 64% (Figure 4).

### 2.4. Protein–Protein Interaction Networks

**Figure 5:**
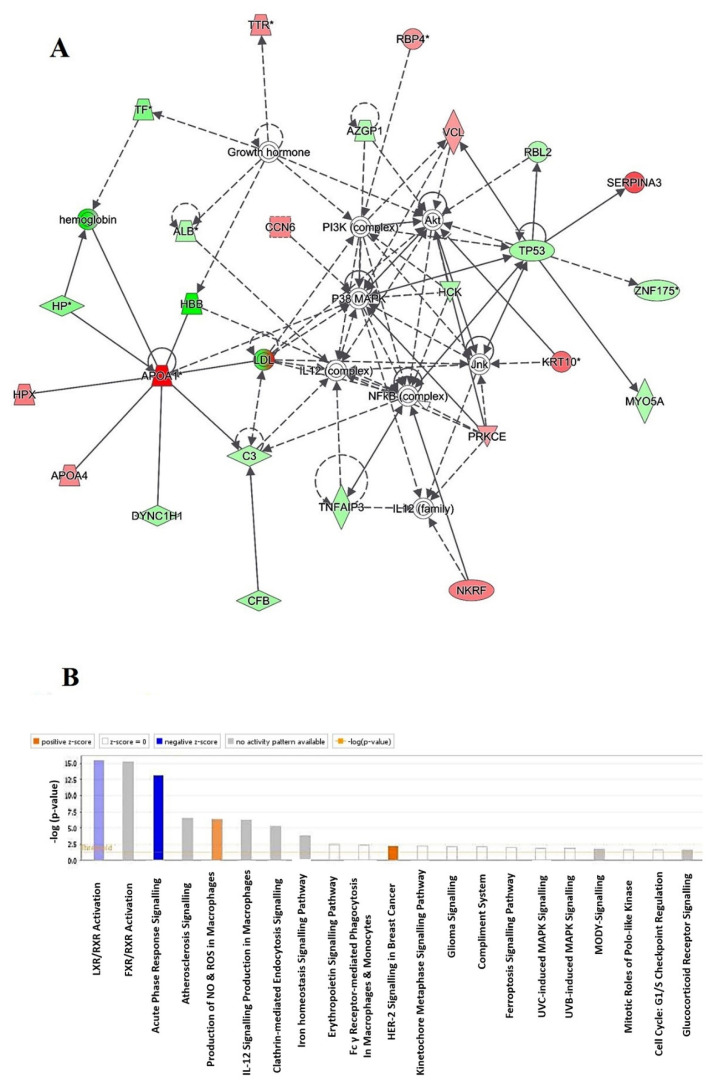
The most enriched interaction network of the differentially expressed proteins in control group as compared with the CUD group. Red nodes indicate upregulated; green nodes indicate downregulated. The central nodes of the pathway are involved in the signaling of the P38 MAPK, NFkB (complex), and Akt, and found to be deregulated between the two groups. Nodes that are not colored are analyzed by IPA and indicate potential targets that were functionally matched with the differentially expressed proteins. Direct molecular interactions are highlighted with solid lines, while indirect interactions are highlighted with dashed lines (**A**). The diagram shows the 21 top canonical pathways ranked by the *p*-values obtained by the IPA (**B**).

Using Ingenuity Pathway Analysis (IPA), the protein–protein interaction analysis was completed for all 121 regulated proteins. The analysis demonstrated that 35 proteins interacted directly/indirectly via protein networks (Figure 5A). The software calculates the best fit score obtained from the input data set of proteins and the biological functions database in order to generate a protein–protein interactions network. The generated network is favorably enriched for proteins with extensive and specific interactions. The interacting proteins are characterized as nodes and their biological relationships as a line. Based on the resulted data, four interaction networks were recognized for the proteins exhibiting variance expression profiles. The highest scoring network (score = 52) (Figure 5, Supplementary Figure S1) incorporated 25 proteins. The proposed highest interaction network pathway was related to free radical scavenging, cellular compromise, and inflammatory response. Alone the top pathways are presented (Figure 5A). Canonical pathways that enriched in current dataset are presented in Figure 5B. The canonical pathways are sorted down to decreasing log (*p*-value) of enrichment. The most interesting enriched canonical pathways included liver X receptors/retinoid X receptor (LXR/RXR) activation (11% overlap, *p*-value: 3.7 × 10^−16^), farnesoid X receptors/retinoid X receptor activation (10.7% overlap, *p*-value: 5.78 × 10^−16^), acute phase response signaling (7.4% overlap, *p*-value: 7.15 × 10^−14^), atherosclerosis signaling (5.6% overlap, *p*-value: 3.87 × 10^−7^), and production of nitric oxide (NO) and reactive oxygen species (ROS) in macrophages (4.3% overlap, *p*-value: 4.24 × 10^−7^). More details about the identified canonical pathways are shown in supplementary files (Supplementary Figure S1).

### 2.5. Subcellular and Functional Characterization of the Differentially Expressed Proteins

**Figure 6:**
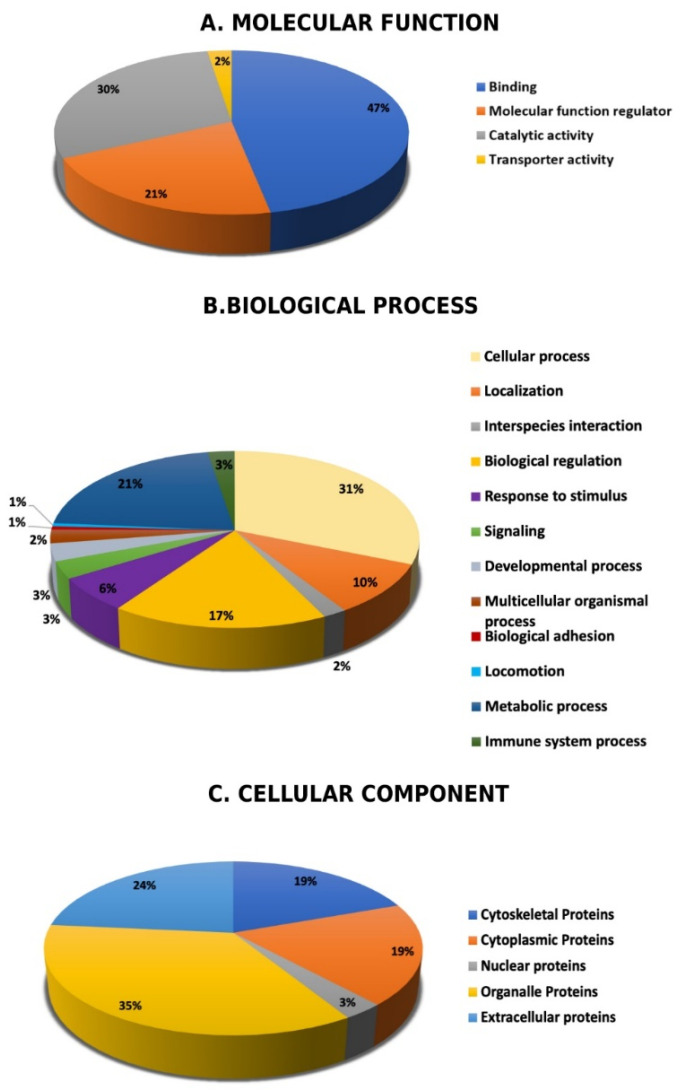
Comparative analysis (%) of identified proteins of interest classified into different groups according to their molecular function (**A**), biological process (**B**), and cellular components (**C**).

Following MS analysis, all 121 identified proteins between the CUD and control samples were subjected to the PANTHER classification system (http://www.pantherdb.org, accessed on 1 February 2021). The classification was performed according to their molecular function (Figure 6A), biological process (Figure 6B), and cellular component (Figure 6C). The main functional categories recognized were binding proteins (47%), catalytic activity (30%), and molecular function regulatory proteins (21%). Further, the identified proteins were located in the organelle region (35%), extracellular space (24%), followed by cytoplasmic and cytoskeletal regions, and each of these two account for (19%). The majority of the identified protein was involved in cellular process, metabolic process and biological regulations.

### 2.6. Immunoblotting Confirmation of Changes in Selected Proteins

**Figure 7:**
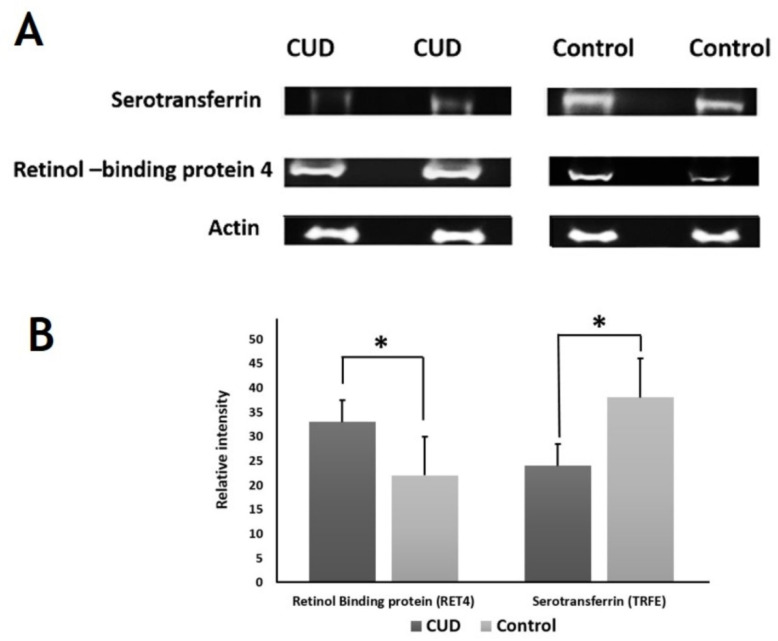
Western blot assay of the chosen proteins identified by 2D-DIGE analysis. Samples from CUD patients and controls were pooled together and used for western blot analysis. The results of Western blot study were similar to the findings of 2D-DIGE (**A**). Graphical analysis of the relative expression data of normalized protein blots between the control and CUD. The presented data are shown as histograms of the mean ± SD and * *p* > 0.05 (**B**).

Immunoblot assay confirmed the expression of the selected proteins that were differentially abundant by 2D-DIGE analysis (Figure 7). The proteins selected for confirmation were serotransferrin and retinol-binding protein 4. Immunoblots revealed that the serum protein expression of serotransferrin and retinol-binding protein 4 were decreased and increased, respectively, in CUD group as compared with control group (*p* ≤ 0.05). To normalize the immunoblot data, β-actin was used in the present study as a housekeeping protein (Figure 7A,B).

## 3. Discussion

### 3.1. Ingenuity Pathway Analysis

#### 3.1.1. LXR/RXR Activation

IPA analysis showed that LXR/RXR is activated in humans chronically exposed to cannabis. Our prior proteomic study showed that LXR/RXR activation is observed in humans exposed to amphetamine for chronic period of time [23]. This indicates that LXR/RXR activation is highly sensitive following exposure to amphetamine and cannabinoids. A microarray study performed IPA analysis showing that THC could induce alterations on the genes, including the LXR/RXR gene, highly affected by lipopolysaccharide in BV-2 microglia cells [31]. This may suggest that LXR/RXR is highly sensitive to THC. Importantly, LXR/RXR activation is linked to signaling pathways, including apolipoproteins such as apolipoprotein AI, for cholesterol metabolism [32]. In the present study, we reported that the serum expression of apolipoprotein AI was increased in patients diagnosed with CUD as compared to healthy control group. This suggests that cannabis-modulated apolipoprotein AI and LXR/RXR may be involved in the metabolism of cholesterol. In addition, LXR activation plays a crucial role in the inhibition of inflammatory responses [33], indicating that LXR/RXR is one of the pathways mediated by cannabinoids to inhibit the formation of inflammatory reactions. For instance, LXR/RXR, PPARα/RXRα, and STAT3 signaling pathways are essential pathways to inhibit the inflammatory reactions [34]. A prior study demonstrated that LXR could play a significant role in induction protective effects against immunological responses-induced by *Mycobacterium tuberculosis* in mice [35]. Therefore, LXR/RXR activation is an efficient therapeutic target to modulate cholesterol metabolism, transport and absorption, inflammatory responses, and immunological reactions. Studies are warranted to explore the beneficial effects of targeting LXR/RXR by cannabinoids to modulate the inflammation, immunological reactions, and cholesterol transport. Further research may investigate the effects of cannabinoids on the diseases through acting on LXR/RXR.

#### 3.1.2. FXR/RXR Activation

IPA analysis showed that FXR/RXR is activated in humans chronically exposed to cannabis. This is in agreement with our previous work showing that FXR/RXR activation is documented in patients diagnosed with amphetamine use disorder [23]. Therefore, FXR/RXR activation can play a critical role in the toxicological effects of abused drugs such as amphetamine and cannabis. The FXR is a bile acid binding site and has a role in the metabolism of lipids and glucose [36]. Our findings reported upregulatory effects on the serum expression of apolipoprotein AI in CUD patients as compared to healthy control group. A previous work highlighted that FXR activation might be a potential strategy for the treatment of hypertriglyceridemia and type 2 diabetes mellitus (T2DM) [37]. This study demonstrated that activation of FXR was associated with reduce plasma concentrations of triglyceride, fasting glucose, and insulin in T2DM rat models. In addition, treatment with a FXR agonist, chenodeoxycholic acid, could reverse the reduction of FXR expression in the liver of T2DM rat models. These findings were supported by another study reporting that hyperlipidemia and hyperglycemia were improved following activation of FXR in diabetic mice models [38]. It is highly recommended to explore the role of cannabinoids on modulating hyperglycemia and hyperlipidemia through modulating FXR/RXR pathways in humans. Further work should study the effects of cannabinoids on the diseases through acting on FXR/RXR.

#### 3.1.3. Acute Phase Response Signaling

In our IPA analysis, we found that acute phase response signaling is stimulated in cannabis users. The acute phase proteins, including haptoglobin, alpha-1-antitrypsin, and complement factors, are proteins that are changed in response to the inflammatory cytokines [39,40]. Acute phase responses are correlated to various diseases, including immunological diseases [41]. The acute phase response signaling was the most interesting enriched canonical pathway involved in patients with amphetamine use disorder as shown in our previous proteomic study [23]. Interestingly, THC exposure was found to increase the mortality in mice infected with *Legionella pneumophila* at least in part by altering the acute phase responses of proinflammatory cytokines, an effect was not observed with cannabinol and cannabidiol as well as a synthetic cannabinoid, CP 55,940 [42]. This suggests that psychoactive cannabinoids might be more likely to modulate the acute phase responses of inflammatory biomarkers. However, cannabidiol and its synthetic analogs have been reported to exert anti-inflammatory and antioxidant effects [18]. CBD is a negative allosteric modulator of cannabinoid receptor 1 (CB1) [43]; moreover, CBD was reported to behave inverse agonist properties to CB2 receptor indicating that CBD-mediated anti-inflammatory effects through modulating CB1 and 2 receptors [44]. The anti-inflammatory properties were also documented with psychoactive cannabinoids [20]. Therefore, it is critical to elucidate the role of psychoactive and non-psychoactive compounds in modulating acute phase proteins.

#### 3.1.4. Atherosclerosis Signaling

In our study, atherosclerosis signaling has been found to be modulated in CUD patients. Atherosclerosis signaling was one of the top canonical pathways that are involved in protein–protein interactions in amphetamine use disorder patients [23]. Note that apolipoprotein AI and inflammatory pathways interact with atherosclerosis signaling [45]. A previous review work discussed that THC might attenuate the plaques, generated from atherosclerosis, through modulating CB2 receptors [46]. Additionally, activation of CB1 receptors in the brain might be a therapeutic strategy to prevent ischemic stroke. Importantly, 2-arachidonoylglycerol (2-AG) and palmitoylethanolamide (PEA) are endocannabinoids that were found to attenuate the acute complication of atherosclerosis such as myocardial ischaemia in isolated rat hearts [47]. These effects were determined by measuring the activities of cardiac creatine kinase (CK) and lactate dehydrogenase. The beneficial effects of 2-AG and PEA on myocardial ischemia were abolished following exposure to a CB2 receptor antagonist, SR144528, indicating that the endocannabinoids might play a vital role in preventing the acute complications of atherosclerosis through acting on CB2 receptors. The significant role of CB2 receptors in preventing the myocardial ischemia was further supported by a study showing that a CB1/2 receptors agonist (WIN55212) was able to reduce the infraction size, an effect abolished with a selective CB2 receptor antagonist (AM630) but not with a selective CB1 antagonist (AM251). Moreover, treatment with a low dose of THC could reduce the progression of atherosclerosis in apolipoprotein E knockout mouse model [48]. This effect was abolished following exposure to a CB2 receptor antagonist. However, smoking cannabis with uncontrolled quantity of cannabinoids may induce adverse effects on the cardiovascular system [49]. These findings provide information about the potential therapeutic values of using cannabinoids for the treatments of atherosclerosis-related diseases.

#### 3.1.5. Production of Nitric Oxide (NO) and Reactive Oxygen Species (ROS) in Macrophages

In our IPA analysis, we reported that NO and ROS production is significantly changed in CUD patients. Studies found that both ROS pathways/production are modulated following exposure to abused drugs [18,50,51]. These data are in agreement with our proteomic work demonstrating that production NO and ROS in macrophages was one of the top canonical pathways that are involved in protein–protein interactions in amphetamine use disorder patients [23]. Importantly, both CB1 and CB2 receptors are key proteins in regulating the productions of ROS and inflammatory cytokines by macrophages [52]. Interestingly, it was shown that activation of CB1 receptor mediated proinflammatory responses by macrophages via increased ROS production in part by inducing p38-mitogen-activated protein kinase phosphorylation [52]. This effect was attenuated through activating Ras-related protein 1, an effect mediated by CB2 receptor pathway. It is found that CBD exhibited potential antioxidant effects through direct and indirect pathways [18]. These pathways include modulating proteins such as CB1 and 2 receptors, antioxidant enzymes, adenosine A_2A_ receptors, and other proteins. Therefore, it is recommended to test the effectiveness of CBD against many diseases associated with oxidative stress. CBD has better safety profile against psychoactive cannabinoids such as THC. Additionally, both THC and CBD could induce neuroprotective effects due to their potential antioxidant properties [53]. Alternatively, a synthetic cannabinoid exposure showed the ability to attenuate the production of NO in chondrocytes treated with IL-1 [54]. This study was supported by another study showing that a synthetic cannabinoid inhibited lipopolysaccharide-induced NO release in macrophages, an effect mediated by CB2 receptor pathway [55]. Cannabichromene, a cannabinoid TRPA1, reduced NO production by macrophages and attenuated colitis of murine [56]. Moreover, THC and CBD attenuated NO production in macrophages exposed to lipopolysaccharide, and the study found that THC was more potent than CBD in reducing the NO production [57]. Furthermore, THC exhibited ability to attenuate the gene expression of inducible NO synthase enzyme through modulating nuclear factor-κB (NF-κB) pathway in macrophages treated with lipopolysaccharide [58]. Taken together, cannabinoids might be potential compounds in modulating NO and ROS production by macrophages in various diseases.

### 3.2. Selected Proteins

Our work provides clinical understanding about the serum proteomic profiling in patients diagnosed with CUD. We found that there are significant alterations in the serum proteins expression and these proteins have been found to be essential in inflammations, protein binding, acute phase reactions, metabolic pathways, and other pathways. Moreover, these proteins have been found to be involved in oxidative stress, thyroid diseases, Alzheimer’s diseases, and lipid disorders. Our data indicate that cellular processes and cellular anatomy are highly affected by cannabis. For this study discussion, we selected proteins that either highly significant altered (*p* values) or changed at different locations in CUD patients as compared with control group.

#### 3.2.1. Albumin

Our study investigated the serum expression of proteins that are highly involved in drug binding, including albumin [59]. Our work revealed that the serum expression of albumin was decreased in CUD patient as compared with control group. As albumin occupies high amount human serum proteins, the expression level of this protein is critical in patients who have developed other diseases and taken certain drugs. An important note that certain antipsychotic, antihypertensive, antiepileptic, antidepressant, antibiotic, and other classes of drugs have high protein binding properties and have narrow therapeutic windows [60]. Therefore, comorbidity of CUD with other diseases/disorders may result in toxicological effects of drugs that were used to treat these diseases or disorders. It is recommended here that CUD patients should be carefully monitored when they take other medicines. Drug–drug interaction between cannabinoids and other classes of drugs has been previously reported [61,62].

#### 3.2.2. Haptoglobin

Regarding the serum haptoglobin level, our study reported a controversial result regarding the circulatory serum levels of haptoglobin in the CUD patients as compared with the control group. It is noteworthy that there is a correlation between haptoglobin and the inflammation process [63,64]. Cannabinoids were found to exert ant-inflammatory effects in animal models [65,66]. Haptoglobin was found to exert a protective effect against oxidative stress induced by an increase in the level of the hemoglobin in pre-clinical models [67]. Moreover, a proportional correlation was observed between haptoglobin and the levels of inflammatory cytokines [68]. In our study, we demonstrated that haptoglobin serum expression was upregulated in some locations and downregulated in other locations in the CUD group as compared with the control group. This differential expression of haptoglobin may result from post-translational modifications, cleavage by enzymes, or different protein species presence. Importantly, THC has been reported to induce oxidative stress, an effect associated with decreased antioxidant parameters [69]. However, CBD was found to produce antioxidant effect in neuronal cells [70,71]. Notably, exposure to *C. sativa* for 30 days resulted in a reduction in the total antioxidant capacity, an effect associated with an increase in the levels reactive oxygen species in male albino rats [72]. More research is required to explore the role of serum haptoglobin level in humans exposed to cannabis and its applications in medical sciences.

#### 3.2.3. Apolipoprotein A-I

Our study found that serum circulatory levels of apolipoprotein A-I is highly abundant in CUD patients as compared with control group. Note that cholesterol levels and lipid metabolism are highly regulated by apolipoprotein A-I. A study reported that Apolipoprotein A-I interacted with high density lipoprotein particles [73]. Gene therapy using apolipoprotein A-I was shown to induce protective effects against lipid disorders [74]. A prior study found that cannabis exposure is associated with weight loss and reduced body mass index in humans [75]. Importantly, it was also found that cannabinoids could induce anorexia in part through acting on cannabinoid receptors [76]. Therefore, cannabis use may lead to an improvement in lipid metabolism and anorexia effects. These effects provide hope to develop novel therapeutic agents from *C. sativa* for the treatment of the lipid diseases. This is in an agreement with previous studies showing that cannabinoids improved heart diseases [77] suggesting the involvement of apolipoprotein A-I in this effect. Alternatively, prior studies found that apolipoprotein A-I had ability to reduce the beta amyloid accumulation [78,79]. This effect may lead to beneficial consequences against Alzheimer’s disease. It is critical to mention here that THC and CBD might be potential compounds for prevention and treatment Alzheimer’s disease symptoms [80]. Moreover, low levels of apolipoprotein A-I in the serum was observed in schizophrenic patients [81]. CBD may have therapeutic effects against schizophrenia [82]. However, studies found that THC exposure was associated with psychosis and schizophrenia [83,84]. The pharmacological effects of cannabis constituents against schizophrenia and Alzheimer’s disease should be further investigated.

#### 3.2.4. Type I and Type II Keratins

Keratin is an essential component that is involved in the epithelial lining. Keratins have protective functions and provide structure to the epithelium [85]. The keratin is a protein that is a fibrous structure and localized in nails, hair, epithelial cells of the skin outer layer, and others [85]. Our study revealed that type I and type II keratins are highly abundant proteins in the serum of CUD compared with control group. Studies found that keratins are essential proteins in cell growth, differentiation, and proliferation [86,87,88]. In addition, they provide mechanical integrity as protection against external stress [89]. They also have cycloprotection properties against non-mechanical stresses [90]. These proteins have an additional role in the digestive system [91]. It is critical to figure out which constituents (cannabinoids vs. non-cannabinoids) in *C. sativa* are responsible for increasing both types (type I and type II) of keratins in the circulatory system in individuals who are chronically exposed to cannabis.

#### 3.2.5. Serotransferrin

Serotransferrin is a critical protein to transport the iron from absorption sites or heme degradation to tissues for storage or utilization [92]. In our current study, we showed a downregulation of the serotransferrin serum expression in the of CUD patients as compared with control group. Additionally, serotransferrin expression was decreased in the urine of cannabis users [93], suggesting that cannabis users have downregulation in serotransferrin in the serum and urine. It is important to consider that a previous proteomic study found that serotransferrin was decreased in the lungs of smokers compared with control group [94]. However, the serum serotransferrin was found to be reduced in amphetamine use disorder patients [23]. This suggests that chronic exposure to abused drugs induces dysregulation in iron and heme balance.

#### 3.2.6. Transthyretin

Transthyretin is an important protein to transport the thyroid hormone, thyroxine, and retinol-binding protein [95]. In this study, we found that there is an upregulation of the serum transthyretin expression in the of CUD patients as compared with control group. In addition, previous studies found that the accumulation or mutations of transthyretin was associated with amyloid diseases such as senile familial amyloid polyneuropathy, systemic amyloidosis, and familial amyloid cardiomyopathy [95]. However, transthyretin showed ability to bind to beta amyloid attenuating beta amyloid aggregation [96,97], which has beneficial consequences against Alzheimer’s disease. Therefore, it is recommended to explore the role of cannabis constituents in modulating transthyretin as a potential biomarker that is involved in many diseases.

#### 3.2.7. Tumor Necrosis Factor Alpha-Induced Protein 3

Tumor necrosis factor alpha-induced protein 3 (TNFAIP3) was found to regulate the activity of NF-κB, especially through the receptor of TNF-alpha [27]. Importantly, NF-κB was found to be increased in the states of inflammation and activated immune cells [98]. Moreover, TNFAIP3 was found to be a negative feedback mechanism for NF-κB activation [99]. Note that a reduction in the expression of TNFAIP3 is a predictor for inflammation and increased NF-κB expression [100]. For instance, it has been found that the gene expression of TNFAIP3 was decreased in peripheral blood mononuclear cells of rheumatoid arthritis patients as compared with healthy control [101]. Moreover, the gene expression of TNFAIP3 was also reduced in peripheral blood mononuclear cells of patients with psoriasis vulgaris [102]. This study found that the gene expression level of TNFAIP3 was negatively linked to the severity of the disease. However, increased TNFAIP3 expression was linked to low survival rate of esophageal squamous cell carcinoma in a non-cancerous esophageal cell line [103]. We reported here that TNFAIP3 was less abundant protein in the circulatory serum of CUD patients as compared with control group.

#### 3.2.8. Inactive Tyrosine Protein Kinase PEAK1

Inactive tyrosine protein kinase PEAK1 is an encoded gene and involved in the cellular response inside the cells following the activation of tyrosine kinase receptor [104]. We here found that inactive tyrosine protein kinase PEAK1 is less abundant in CUD patients as compared with healthy controls. Importantly, increased PEAK1 has been linked to the progression and metastasis of breast cancer [104,105]. We suggest that further investigation of the modulatory role of cannabinoids in the progression of breast cancer is required. PEAK1 has been found to regulate the responses of transforming growth Factor β in breast cancer models [104]. Moreover, overexpression of inactive tyrosine protein kinase PEAK1 can modulate anus kinase-2 and extracellular signal-regulated kinase-1/2, which was involved metastasis of tumors in lung cancers [106]. Previous and our findings suggest that future directions may explore the potential role of cannabis plants in cancer research focusing on the PEAK1 pathways.

## 4. Materials and Methods

### 4.1. Ethical Approval and Participate Consent

The IRB committees at the Eradah Complex for Mental Health (Riyadh, Saudi Arabia) and College of Medicine-King Saud University reviewed and approved all procedures and protocols. This study was performed according to the rules of the Declaration of Helsinki 1975 and later amendments. The written consents were acquired from all individuals involved in the study. This study was conducted at the Proteomics Unit, Obesity Research Center, College of Medicine and King Khalid University Hospital, Medical City, King Saud University, Riyadh, Saudi Arabia.

### 4.2. Study Design and Selection Criteria

Twenty male subjects were involved in two different groups in this study: CUD and healthy control. Ten subjects diagnosed with CUD (age of 30.4 ± 4.36 years) were enrolled at the Eradah Complex for Mental Health (Riyadh, Saudi Arabia) and compared with a control group containing 10 healthy individuals (age of 24.7 ± 3.63 years). To perform the power analysis and determine the least possible number of required biological replicates, we used Progenesis SameSpots software (Nonlinear Dynamics, Newcastle, UK). The diagnosis for CUD was performed according to the Diagnostic and Statistical Manual of Mental Disorders guidelines (DSM-5) [107]. The included participants have no history of blood disorders, diabetes mellitus, obesity, psychosis, renal diseases, or any other infectious diseases. Clinical and demographic information is shown in Table 1. The control group demographic information and gel scanning are used from our previous study [23] with respect of experimental procedures and timing. The CUD group tested positive for cannabinoids only without any detection of other abused drugs at the time of blood collection. Blood samples were collected, centrifuged for ten minutes at 1000× *g*. The resulting serum samples were aliquoted and stored at −80 °C for proteomic analysis.

### 4.3. Serum Protein Extraction

Proteins were extracted from the serum samples via centrifugation (5 min, 12,000× *g*) as described previously [23]. The depletion of high-abundance serum proteins (i.e., albumin, IgG) was achieved using Depletion SpinTrap for Albumin and IgG (GE Healthcare, Chicago, IL, USA) following the manufacturer’s instructions. Further, the remaining proteins were extracted by the TCA/acetone method [108]. The depleted samples were mixed with ice-cold acetone containing 10% *w/v* TCA (1:4), and the mixture was vortexed for 15 s to ensure uniform mixing. Next, the mixture was incubated overnight at −20 °C for protein precipitation. After incubation, the tubes were centrifuged for 15 min at 4 °C at a speed of 12,000× *g*, and the pellet was solubilized in labeling buffer (7 M of urea, 2 M of thiourea, 30 mM of Tris-HCl, 4% CHAPS, pH 8.5). After that, the concentration of protein samples was determined in triplicate employing the 2D-Quant Kit (GE Healthcare, Chicago, IL, USA).

### 4.4. Fluorescence Labeling of Samples with CyDyes and 2-Dimensional Difference in Gel Electrophoresis (2D-DIGE)

Fifty micrograms of protein from each sample of both the CUD and control groups was labeled with 400 pmol of Cy3 and Cy5 dyes. Then, the internal standard was prepared by mixing an equal amount of all samples after pooling and labelling with Cy2. A dye swapping strategy was employed during labelling in order to avoid any dye-specific bias (Supplementary Table S1). 1st-dimension analytical GE followed by 2nd-dimension sodium dodecyl sulfate (SDS)-polyacrylamide GE (SDS-PAGE) were implemented on 12.5% fixed gels as described in previous studies [23,109]. Further, the 2D-DIGE gels were scanned using the Typhoon 9400 scanner (GE Healthcare, Chicago, IL, USA) where specific excitation/emission wavelengths were used (488/520 nm) for Cy2, (532/580 nm) for Cy3, and (633/670 nm) for Cy5.

### 4.5. Statistical Analysis

Progenesis SameSpots software (v2.0, Nonlinear Dynamics, Newcastle, UK) was used to analyze the 2D-DIGE gel images. The image analysis was done by an automated spot detection and comparison method between the samples of CUD and control groups. Although the automatic analysis was completed to detect all the spots across all the 10 gels, each selected spot was manually edited and verified wherever necessary. The differentially expressed spots were identified by normalized volumes. The normalized volume of each spot on each gel was calculated from Cy3 (or Cy5) to Cy2 spot volume ratio using the software. To generate normal distributed data, log transformation of the spot volumes was done by the software. To calculate statistically significant differences between the two groups, one-way ANOVA was used and *p* < 0.05 was considered statistically significant. A cut-off ratio ≥1.5-fold was considered significant. A pre-filtration and manual check have been done on all spots before testing the statistical differences. In statistical analysis, the normalized spot volumes were applied instead of intensities of the spots. Any spots fulfil the above statistical criteria was analyzed by MS.

### 4.6. Protein Identification with Mass Spectrometry

Coomassie-stained gel spots from a preparatory gel were washed then digested according to methods described previously [23,109,110]. To describe briefly, total protein (1 mg) was obtained from a pool of equal protein amounts of the 20 serum samples (10 CUD and 10 control). This sample was denatured in lysis buffer and then mixed in a rehydration buffer. Then, the proteins samples were separated by first and second dimensions with the same conditions in the DIGE section. Then, the gels were fixed in 40% (*v*/*v*) ethanol containing 10% (*v*/*v*) acetic acid (overnight) and then washed (3×, 30 min each, ddH_2_O). The gels were incubated (1 h, 34% (*v*/*v*) CH3OH containing 17% (*w*/*v*) ammonium sulphate and 3%(*v*/*v*) phosphoric acid) prior to the addition of 0.5 g/L Coomassie G-250. After 5 days, the stained gels were briefly rinsed with Milli-Q water and stored until the spots could be picked and identified by MS. Digestion was performed by adding 15 µL of (20 ng ice-cold trypsin solution in 25 mM NH_4_HCO_3_, 5 mL CH_3_CN, 5 mL distilled water) and incubated 20 min at 4 °C, and digestion continued overnight at 37 °C. To extract the peptides, 1 µL of 1% Trifluoracetic acid was added on the gel pieces and placed in vortex incubator for mass spectrometric analysis (1 h, 400 rpm, 25 °C).

After that, a mixture of tryptic peptides (1 μL) was formed from each protein and spotted onto a MALDI target (384 MTP Anchorchip; 800 μm Anchorchip; Bruker Daltonics, Bremen, Germany). MALDI-MS spectra were obtained with UltraflexTerm time-of-flight (TOF) MS equipped with a LIFT-MS/MS device (Bruker Daltonics, Bremen, Germany) at a reflector (voltages of 21 kV) and detector (voltages of 17 kV), as described previously [111,112,113]. PMFs were calibrated against peptide calibration standard II (Bruker Daltonics, Bremen, Germany). The PMFs were assessed with Flex Analysis software (v2.4, Bruker Daltonics, Bremen, Germany). MS data were interpreted with BioTools v3.2 (Bruker Daltonics, Bremen, Germany). The peptide masses were searched against the Mascot search algorithm (v2.0.04, updated on 9 May 2019; Matrix Science Ltd., Bremen, UK). The identified proteins were screened for a Mascot score >56 and *p* < 0.05.

### 4.7. Network Pathway and Functional Analysis

The IPA Software program (Version: 42012434, Ingenuity Systems, Redwood City, CA, USA, http://www.ingenuity.com, accessed on 2 February 2021) was used to analyze the identified proteins and to annotate them with related functions and pathways. The annotations involved overlaying the proteins with their most significant networks and biochemical pathways based on previous publications on the proteins. The identified proteins were classified into different categories according to their biological process, cellular components, and molecular function using protein analysis through evolutionary relationships (PANTHER) classification system (http://www.pantherdb.org, accessed 1 February 2021).

### 4.8. Immunoblotting

Immunoblotting assay was performed in the current study to further confirm the findings of the proteomic study. Two differential abundance proteins with statistically significant were chosen and determined by immunoblotting. Primary monoclonal antibodies against transferrin (mouse, cat # SC-365871), retinol-binding protein (RBP, mouse, cat # SC-69795), and β-actin (goat, N-18, cat # SC-1616) were bought from Santa Cruz Biotechnology (Santa Cruz, TX, USA). One-dimensional discontinuous slab gel electrophoresis (12% sodium dodecyl sulfate (SDS)-polyacrylamide gel) was used to separate an equal amount of protein from each sample (50 μg). A mini trans-blot electrotransfer cell (BioRad, California, CA, USA) was employed to transfer proteins from the run gels to an Immobilon-P, polyvinylidene difluoride (PVDF) transfer membrane (Millipore, Massachusetts, MA, USA) To test the efficiency of the transfer, the membranes were stained with Ponceau-S. Subsequently, the membranes were blocked with tris-buffered saline (TBS)-containing 5% fat-free milk (FFM), for one hour at room temperature, and then the membranes were rinsed three times with TBS-T in 10 mM Tris–HCl, 150 mM NaCl, 0.1% Tween 20 buffer. After rinsing, the membranes were incubated with the selected primary antibodies at dilution of (1:200) using a blocking buffer. Membranes were then incubated with the matched immunoglobulin G (IgG)-horseradish peroxidase (HRP)-conjugated secondary antibody, and the enhanced chemiluminescence (ECL, Thermo Fisher Scientific, Massachusetts, MA, USA) was used to detect the immunoreactive bands. These bands were visualized by scanning with Sapphire Biomolecular Imager (Azure Bio systems, Dublin, OH, USA) and digitalized via the image analysis software Sapphire Capture system (Azure Biosystems, Dublin, OH, USA).

## 5. Conclusions

Our findings provide clinical insight about the potential effects of cannabis abuse on the circulatory protein expression. These proteins are highly involved in different applications and diseases/disorders. The present study highlighted that drug discovery research can further investigate the effects of cannabis ingredients on immunological and inflammatory responses as well as the diseases involved, e.g., atherosclerosis. This will prove a novel direction to discover and develop potential compounds for the prevention or treatments of these diseases. These researches might target acute phase proteins, NO and ROS pathways, atherosclerosis signaling, or LXR/RXR and FXR/RXR pathways. One of applications obtained from the current work is the drug–drug interaction since our present study showed that the serum expression of albumin, which is a major serum protein binding, is decreased in CUD patients. Our study also found that cannabis abuse might modulate several diseases and disorders. However, the quantity of cannabis inside the humans were not controlled; therefore, this hypothesis needs more investigations with controlled doses. In addition, cannabis include cannabinoids and non-cannabinoids where the cannabinoids are either psychoactive or non-psychoactive. Thus, future studies should further investigate our findings using a specific cannabis constituent. This will provide a clear understanding about the responsible compound for a specific effect. A limitation in our study is the age variation in both groups. More research is required to exclude any age variation and also investigate the serum proteomic profiling of female CUD patients.

## Figures and Tables

**Table 1:** Clinical and demographic information of the CUD and control groups recruited in the present study. HIV: Human immunodeficiency virus; HPC: Hepatitis C virus; TB: Tuberculosis.

	CUD Group	Control Group
Number of patients	10	10
Gender	10 Male, 0 Female	10 Male, 0 Female
Age in years (Mean ± SD)	24.7 ± 3.63	30.4 ± 4.36
Infectious diseases (HIV, HCV, TB)	Negative	Negative
Cannabis dosage form	Smoking	None
Cannabis use history		
2–5 years	4 Patients	None
6–10 years	3 Patients	None
≥11 years	3 Patients	None

**Table 2:** Identified proteins with changes in abundance between CUD and control samples. The table displays average ratio values for control and treated samples with their corresponding levels of fold changes and *p*-values for one-way ANOVA (*p*-value < 0.05) using 2D-DIGE. Analysis type: MALDI-TOF; database: SwissProt; taxonomy: *Homo sapiens*. ^a^ Protein accession number for SWISSPROT Database. ^b^ *p*-Value (ANOVA). ^c^ Ratio between the groups ^d^ Protein expression between the groups.

Sl No:	Spot No ^a^	Accession No	Protein Name	Mascot ID	*p*-Value ^b^ (ANOVA)	Ratio CB/C ^c^	Exp ^d^
**Upregulated Proteins**							
84	990	Q5BJF6	Outer dense fiber protein 2	ODFP2_HUMAN	0.05	2	UP
10	1470	P02647	Apolipoprotein A-I	APOA1_HUMAN	0.01	3.8	UP
25	185	P01011	Alpha-1-antichymotrypsin	AACT_HUMAN	0.02	2.7	UP
79	1627	P02647	Apolipoprotein A-I	APOA1_HUMAN	0.04	2.6	UP
70	1180	Q8TED0	U3 small nucleolar RNA-associated protein 15 homolog	UTP15_HUMAN	0.03	2.6	UP
16	1322	P00739	Haptoglobin-related protein	HPTR_HUMAN	0.01	2.4	UP
81	993	Q7Z398	Zinc finger protein 550	ZN550_HUMAN	0.04	2.4	UP
8	1226	Q9H254	Spectrin beta chain, non-erythrocytic 4	SPTN4_HUMAN	0.01	2.3	UP
5	1797	P13645	Keratin, type I cytoskeletal 10	K1C10_HUMAN	0.007	2.2	UP
42	1260	P02647	Apolipoprotein A-I	APOA1_HUMAN	0.04	2.1	UP
20	1427	Q9BZ29	Dedicator of cytokinesis protein 9	DOCK9_HUMAN	0.02	2.1	UP
58	886	P00738	Haptoglobin	HPT_HUMAN	0.02	2.1	UP
112	676	P02749	Beta-2-glycoprotein 1	APOH_HUMAN	0.05	2	UP
40	1475	Q3L8U1	Chromodomain-helicase-DNA-binding protein 9	CHD9_HUMAN	0.04	2.0	UP
30	334	P02790	Hemopexin	HEMO_HUMAN	0.04	2	UP
46	1705	O15226	NF-kappa-B-repressing factor	NKRF_HUMAN	0.0	2	UP
41	1295	Q969Q6	Serine/threonine-protein phosphatase 2A regulatory subunit B’’ subunit gamma	P2R3C_HUMAN	0.05	2.0	UP
93	1908	P02647	Apolipoprotein A-I	APOA1_HUMAN	0.03	1.9	UP
64	903	O95389	Cellular communication network factor 6	WISP3_HUMAN	0.03	1.9	UP
77	953	P00738	Haptoglobin	HPT_HUMAN	0.04	1.9	UP
99	796	P00738	Haptoglobin	HPT_HUMAN	0.02	1.9	UP
9	1487	P01834	Immunoglobulin kappa constant	IGKC_HUMAN	0.01	1.9	UP
36	1231	P13645	Keratin, type I cytoskeletal 10	K1C10_HUMAN	0.04	1.9	UP
100	987	Q709C8	Vacuolar protein sorting-associated protein 13C	VP13C_HUMAN	0.03	1.9	UP
92	981	P06727	Apolipoprotein A-IV	APOA4_HUMAN	0.04	1.8	UP
96	623	Q92608	Dedicator of cytokinesis protein 2	DOCK2_HUMAN	0.02	1.8	UP
21	1120	O75648	Mitochondrial tRNA-specific 2-thiouridylase 1	MTU1_HUMAN	0.02	1.8	UP
6	1937	Q5T6V5	Queuosine salvage protein	CI064_HUMAN	0.008	1.8	UP
2	1792	P02766	Transthyretin	TTHY_HUMAN	0.005	1.8	UP
76	1865	P02766	Transthyretin	TTHY_HUMAN	0.04	1.8	UP
59	457	Q96M63	Coiled-coil domain-containing protein 114	CC114_HUMAN	0.05	1.7	UP
61	1305	Q12852	Mitogen-activated protein kinase kinase kinase 12	M3K12_HUMAN	0.03	1.7	UP
83	974	P35558	Phosphoenolpyruvate carboxykinase, cytosolic [GTP]	PCKGC_HUMAN	0.05	1.7	UP
115	714	Q02156	Protein kinase C epsilon type	KPCE_HUMAN	0.04	1.7	UP
22	1759	P02766	Transthyretin	TTHY_HUMAN	0.02	1.7	UP
67	1067	Q9NQW7	Xaa-Pro aminopeptidase 1	XPP1_HUMAN	0.03	1.7	UP
80	1659	P02647	Apolipoprotein A-I	APOA1_HUMAN	0.04	1.6	UP
57	1446	Q9BV73	Centrosome-associated protein CEP250	CP250_HUMAN	0.03	1.6	UP
12	1136	O75636	Ficolin-3	FCN3_HUMAN	0.02	1.6	UP
51	1277	O75636	Ficolin-3	FCN3_HUMAN	0.05	1.6	UP
47	1132	Q92896	Golgi apparatus protein 1	GSLG1_HUMAN	0.05	1.6	UP
118	797	P00738	Haptoglobin	HPT_HUMAN	0.04	1.6	UP
44	1962	P13645	Keratin, type I cytoskeletal 10	K1C10_HUMAN	0.04	1.6	UP
23	1193	P04264	Keratin, type II cytoskeletal 1	K2C1_HUMAN	0.02	1.6	UP
87	955	Q5BJF6	Outer dense fiber protein 2	ODFP2_HUMAN	0.05	1.6	UP
39	1784	P02753	Retinol-binding protein 4	RET4_HUMAN	0.04	1.6	UP
56	1669	P02753	Retinol-binding protein 4	RET4_HUMAN	0.02	1.6	UP
35	1473	Q9HBT8	Zinc finger protein 286A	Z286A_HUMAN	0.04	1.6	UP
55	1138	Q6ZN19	Zinc finger protein 841	ZN841_HUMAN	0.02	1.6	UP
105	788	A8TX70	Collagen alpha-5(VI) chain	CO6A5_HUMAN	0.04	1.5	UP
97	771	Q96EH8	E3 ubiquitin-protein ligase NEURL3	LINCR_HUMAN	0.05	1.5	UP
85	1211	P04264	Keratin, type II cytoskeletal 1	K2C1_HUMAN	0.05	1.5	UP
90	913	P02538	Keratin, type II cytoskeletal 6A	K2C6A_HUMAN	0.04	1.5	UP
89	1039	P78332	RNA-binding protein 6	RBM6_HUMAN	0.05	1.5	UP
32	1563	P18206	Vinculin	VINC_HUMAN	0.04	1.5	UP
**Downregulated Proteins**							
88	2217	P68871	Hemoglobin subunit beta	HBB_HUMAN	0.05	−5.0	DOWN
94	2323	P02042	Hemoglobin subunit delta	HBD_HUMAN	0.03	−4.2	DOWN
82	562	P19652	Alpha-1-acid glycoprotein 2	A1AG2_HUMAN	0.04	−3.7	DOWN
95	481	Q5R372	Rab GTPase-activating protein 1-like	RBG1L_HUMAN	0.02	−2.9	DOWN
34	1448	Q6ZNG0	Zinc finger protein 620	ZN620_HUMAN	0.04	−2.6	DOWN
45	456	Q9HAC8	Ubiquitin domain-containing protein 1	UBTD1_HUMAN	0.04	−2.6	DOWN
31	450	P01009	Alpha-1-antitrypsin	A1AT_HUMAN	0.04	−2.5	DOWN
18	385	P02787	Serotransferrin	TRFE_HUMAN	0.02	−2.4	DOWN
7	1082	O43300	Leucine-rich repeat transmembrane neuronal protein 2	LRRT2_HUMAN	0.009	−2.3	DOWN
11	382	P02787	Serotransferrin	TRFE_HUMAN	0.01	−2.2	DOWN
33	853	P00738	Haptoglobin	HPT_HUMAN	0.04	−2.2	DOWN
4	860	P02787	Serotransferrin	TRFE_HUMAN	0.006	−1.9	DOWN
14	865	P00738	Haptoglobin	HPT_HUMAN	0.01	−1.9	DOWN
17	857	Q07065	Cytoskeleton-associated protein 4	CKAP4_HUMAN	0.01	−1.9	DOWN
27	235	P17039	Zinc finger protein 30	ZNF30_HUMAN	0.026	−1.9	DOWN
38	497	P01009	Alpha-1-antitrypsin	A1AT_HUMAN	0.04	−1.9	DOWN
48	1877	P20472	Parvalbumin alpha	PRVA_HUMAN	0.05	−1.9	DOWN
62	1394	Q14204	Cytoplasmic dynein 1 heavy chain 1	DYHC1_HUMAN	0.03	−1.9	DOWN
73	1106	Q8TD57	Dynein heavy chain 3, axonemal	DYH3_HUMAN	0.04	−1.9	DOWN
13	7	P02774	Vitamin D-binding protein	VTDB_HUMAN	0.02	−1.8	DOWN
72	388	P00751	Complement factor B	CFAB_HUMAN	0.03	−1.8	DOWN
3	202	P21580	Tumor necrosis factor alpha-induced protein 3	TNAP3_HUMAN	0.005	−1.7	DOWN
29	661	P01009	Alpha-1-antitrypsin	A1AT_HUMAN	0.04	−1.7	DOWN
43	636	Q99661	Kinesin-like protein KIF2C	KIF2C_HUMAN	0.04	−1.7	DOWN
52	354	O60384	Putative zinc finger protein 861	YS022_HUMAN	0.05	−1.7	DOWN
53	650	P01009	Alpha-1-antitrypsin	A1AT_HUMAN	0.05	−1.7	DOWN
68	423	Q5R372	Rab GTPase-activating protein 1-like	RBG1L_HUMAN	0.03	−1.7	DOWN
71	805	P04637	Cellular tumor antigen p53	P53_HUMAN	0.03	−1.7	DOWN
98	341	Q9P219	Protein Daple	DAPLE_HUMAN	0.05	−1.7	DOWN
104	1465	P17017	Zinc finger protein 14	ZNF14_HUMAN	0.03	−1.7	DOWN
15	864	P01024	Complement C3	CO3_HUMAN	0.01	−1.6	DOWN
24	108	O75121	Microfibrillar-associated protein 3-like	MFA3L_HUMAN	0.02	−1.6	DOWN
26	266	P02768	Albumin	ALBU_HUMAN	0.03	−1.6	DOWN
28	357	P08631	Tyrosine protein kinase HCK	HCK_HUMAN	0.03	−1.6	DOWN
37	548	P02765	Alpha-2-HS-glycoprotein	FETUA_HUMAN	0.04	−1.6	DOWN
54	1037	Q14683	Structural maintenance of chromosomes protein 1A	SMC1A_HUMAN	0.05	−1.6	DOWN
66	1083	Q9UKX3	Myosin-13	MYH13_HUMAN	0.03	−1.6	DOWN
74	579	P02768	Albumin	ALBU_HUMAN	0.04	−1.6	DOWN
86	1054	Q9H792	Inactive tyrosine protein kinase PEAK1	SG269_HUMAN	0.05	−1.6	DOWN
102	702	Q9UM13	Anaphase-promoting complex subunit 10	APC10_HUMAN	0.03	−1.6	DOWN
103	533	P02774	Vitamin D-binding protein	VTDB_HUMAN	0.03	−1.6	DOWN
108	633	P02768	Albumin	ALBU_HUMAN	0.05	−1.6	DOWN
114	537	P02768	Albumin	ALBU_HUMAN	0.05	−1.6	DOWN
120	550	Q8TE73	Dynein heavy chain 5, axonemal	DYH5_HUMAN	0.05	−1.6	DOWN
1	253	Q9H792	Inactive tyrosine protein kinase PEAK1	SG269_HUMAN	0.004	−1.5	DOWN
19	820	Q14585	Zinc finger protein 345	ZN345_HUMAN	0.02	−1.5	DOWN
49	504	Q08999	Retinoblastoma-like protein 2	RBL2_HUMAN	0.05	−1.5	DOWN
50	1484	P02768	Albumin	ALBU_HUMAN	0.05	−1.5	DOWN
60	522	Q9Y473	Zinc finger protein 175	ZN175_HUMAN	0.03	−1.5	DOWN
63	48	P49792	E3 SUMO-protein ligase RanBP2	RBP2_HUMAN	0.03	−1.5	DOWN
65	28	Q14624	Inter-alpha-trypsin inhibitor heavy chain H4	ITIH4_HUMAN	0.03	−1.5	DOWN
69	1213	Q14683	Structural maintenance of chromosomes protein 1A	SMC1A_HUMAN	0.03	−1.5	DOWN
75	215	Q9Y4I1	Unconventional myosin-Va	MYO5A_HUMAN	0.04	−1.5	DOWN
78	528	P02768	Albumin	ALBU_HUMAN	0.04	−1.5	DOWN
91	604	Q6AW86	Zinc finger protein 324B	Z324B_HUMAN	0.04	−1.5	DOWN
101	732	P25311	Zinc-alpha-2-glycoprotein	ZA2G_HUMAN	0.03	−1.5	DOWN
106	584	Q8TD57	Dynein heavy chain 3, axonemal	DYH3_HUMAN	0.04	−1.5	DOWN
107	1688	P20472	Parvalbumin alpha	PRVA_HUMAN	0.04	−1.5	DOWN
109	709	Q9NZM1	Myoferlin	MYOF_HUMAN	0.05	−1.5	DOWN
110	198	P02768	Albumin	ALBU_HUMAN	0.05	−1.5	DOWN
111	743	P01009	Alpha-1-antitrypsin	A1AT_HUMAN	0.05	−1.5	DOWN
113	556	P20929	Nebulin	NEBU_HUMAN	0.05	−1.5	DOWN
116	588	Q96SZ6	Mitochondrial tRNA methylthiotransferase CDK5RAP1	CK5P1_HUMAN	0.04	−1.5	DOWN
117	275	Q9Y473	Zinc finger protein 175	ZN175_HUMAN	0.05	−1.5	DOWN
119	768	O76041	Nebulette	NEBL_HUMAN	0.04	−1.5	DOWN
121	549	P51508	Zinc finger protein 81	ZNF81_HUMAN	0.03	−1.5	DOWN

## Data Availability

All data generated or analyzed in the current study are included in this article. The mass lists obtained from the MALDI-TOF-MS analysis of the 121 spots in this study are openly available in FigShare at doi:10.6084/m9.figshare.15131253.

## Supplementary Materials

The following are available online, Figure S1: Pathways and canonical pathways identified in the IPA functional analysis, Figure S2: Gel images, Figure S3: Example of the full western blots (not truncated), Table S1: Experimental design, Table S2: Mass spectrometry list of significant differentially abundant proteins, Table S3: List of 25 proteins with accession numbers depicted in IPA network pathway, and Table S4: List of proteins with accession numbers for the top 5 canonical pathways.

## Sample Availability

Samples of the compounds are not available from the authors.
