# Supplementary material for: Serum Proteomic Analysis of Cannabis Use Disorder in Male Patients
Source: Molecules. 2021 Sep 1;26(17):5311. doi: 10.3390/molecules26175311 (PMC8434053; doi:10.3390/molecules26175311)
Supplement: Supplementary file 1 [file molecules-26-05311-s001.zip › molecules-1295170-supplementary.pdf]

**Supplementary data:**

**Figure S1:** Pathways and canonical pathways identified in the IPA functional analysis.

**Top Canonical Pathways**

| Name | p-value | Overlap |
|---|---|---|
| LXR/RXR Activation | 3.70E-16 | 11.0 % 13/118 |
| FXR/RXR Activation | 5.78E-16 | 10.7 % 13/122 |
| Acute Phase Response Signaling | 7.15E-14 | 7.4 % 13/176 |
| Atherosclerosis Signaling | 3.87E-07 | 5.6 % 7/126 |
| Production of Nitric Oxide and Reactive Oxygen Species in Macrophages | 4.24E-07 | 4.3 % 8/188 |

**Top Networks**

| ID | Associated Network Functions | Score |
|---|---|---|
| 1 | Free Radical Scavenging, Cellular Compromise, Inflammatory Response | 52 |
| 2 | Cell-To-Cell Signaling and Interaction, Hematological System Development and Function, Immune Cell Trafficking | 26 |
| 3 | Cancer, Hematological Disease, Immunological Disease | 17 |
| 4 | Cell Death and Survival, Skeletal and Muscular Disorders, Cancer | 11 |

**Figure S2:** Gel images (n=10) of the study

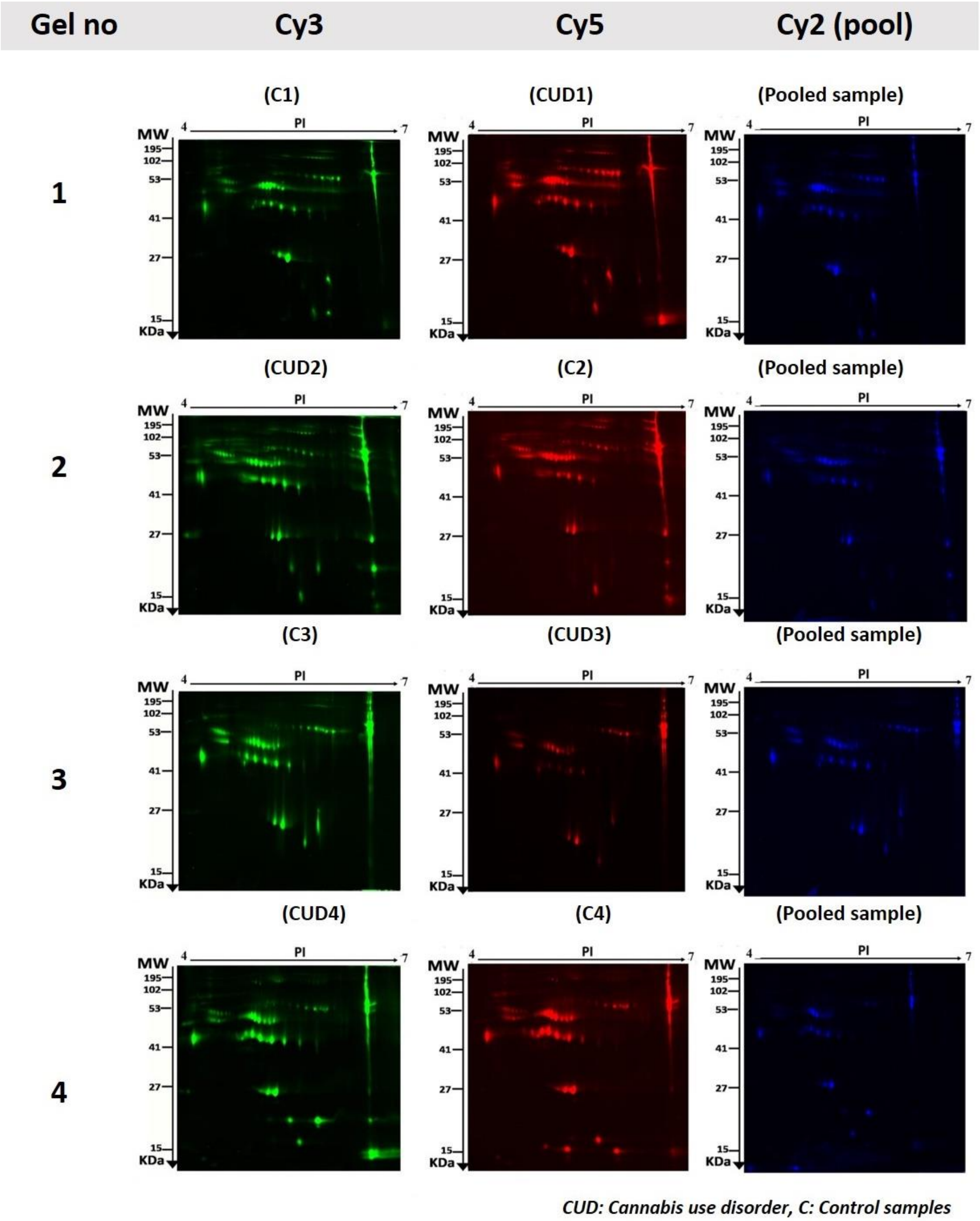

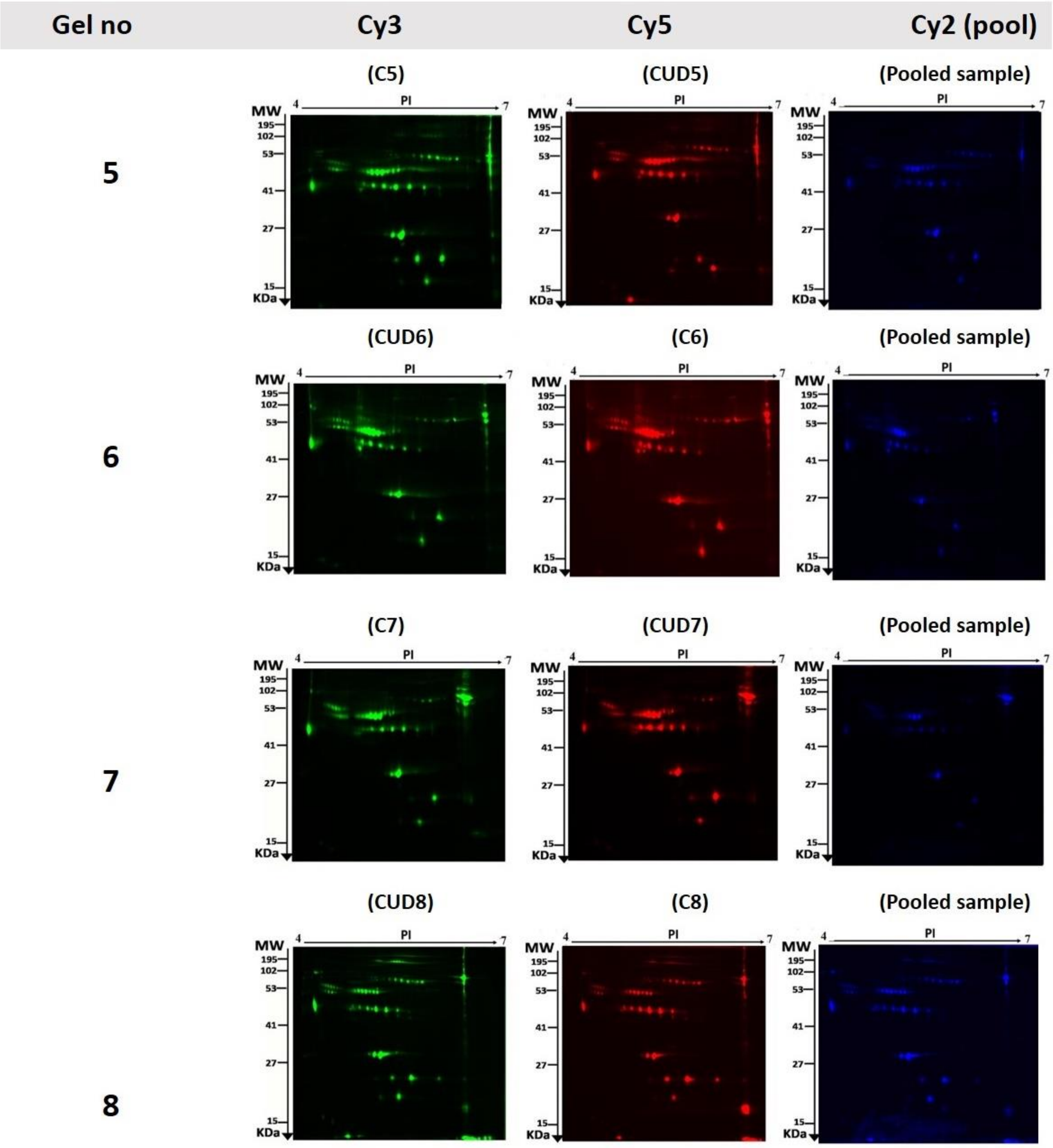


*CUD: Cannabis use disorder, C: Control samples*

| Gel no | Cy3 | Cy5 | Cy2 (pool) |
| --- | --- | --- | --- |
| 9 | (C9) | (CUD9) | (Pooled sample) |
| | 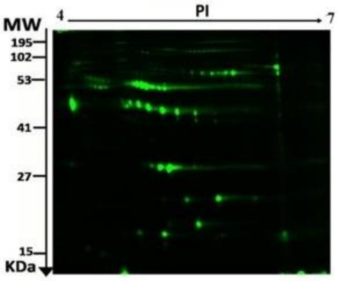 | 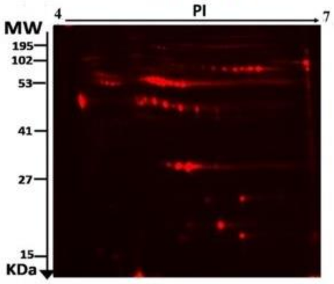 | 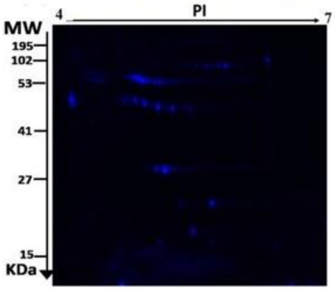 |
| 10 | (CUD10) | (C10) | (Pooled sample) |
| | 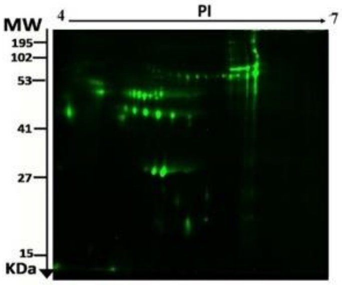 | 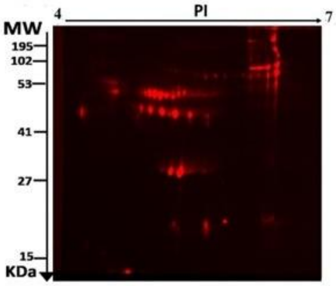 | 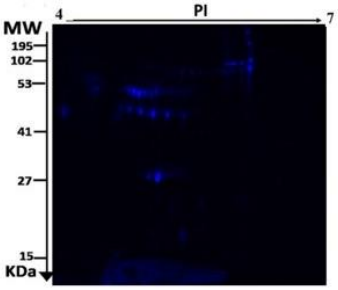 |

*CUD: Cannabis use disorder, C: Control samples*

**Figure S3:** Example of the full western blots (not truncated)

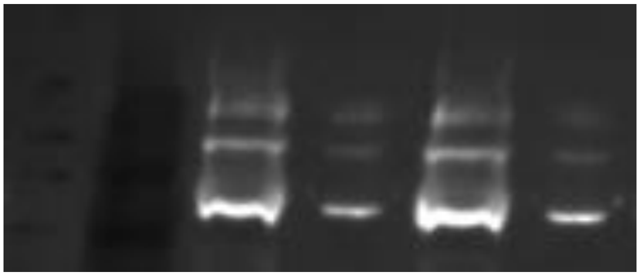

**Table S1:** Dye-switching strategy applied during labeling to avoid dye-specific bias. A total of 20 patient samples were run on 10 2D-PAGE gels. Samples were labeled randomly with Cy3 and Cy5, and a pooled sample was used as an internal standard and was stained with Cy2 (CUD-Cannabis use disorder, C- Control).

| Gel | Cy3 | Cy5 | Cy2 |
|---|---|---|---|
| 1 | C1 | CUD1 | Pooled sample |
| 2 | CUD2 | C2 | Pooled sample |
| 3 | C3 | CUD3 | Pooled sample |
| 4 | CUD4 | C4 | Pooled sample |
| 5 | C5 | CUD5 | Pooled sample |
| 6 | CUD6 | C6 | Pooled sample |
| 7 | C7 | CUD7 | Pooled sample |
| 8 | CUD8 | C8 | Pooled sample |
| 9 | C9 | CUD9 | Pooled sample |
| 10 | CUD10 | C10 | Pooled sample |

**Table S2:** List of significantly differentially abundant proteins between CUD patients and control samples identified in samples using 2D-DIGE coupled with MS. Protein name, accession number, Mascot score, MS coverage (64%), protein MW, and pI values according to Uniprot database are listed.

| Sl no: | Spot No[a] | Accession No[b] | Protein Name | MASCOT ID | Pi[c] | MW[d] | Cov% | Score[e] |
|---|---|---|---|---|---|---|---|---|
| 1 | 253 | Q9H792 | Inactive tyrosine-protein kinase PEAK1 | SG269_HUMAN | 6.46 | 195039 | 15 | 56 |
| 2 | 1792 | P02766 | Transthyretin | TTHY_HUMAN | 5.52 | 15991 | 69 | 92 |
| 3 | 202 | P21580 | Tumor necrosis factor alpha-induced protein 3 | TNAP3_HUMAN | 8.61 | 91837 | 17 | 60 |
| 4 | 860 | P02787 | Serotransferrin | TRFE_HUMAN | 6.82 | 79280 | 21 | 143 |
| 5 | 1797 | P13645 | Keratin, type I cytoskeletal 10 | K1C10_HUMAN | 5.13 | 59020 | 22 | 96 |
| 6 | 1937 | Q5T6V5 | Queuosine salvage protein | CI064_HUMAN | 5.61 | 39460 | 31 | 57 |
| 7 | 1082 | O43300 | Leucine-rich repeat transmembrane neuronal protein 2 | LRRT2_HUMAN | 8.27 | 59950 | 15 | 62 |
| 8 | 1226 | Q9H254 | Spectrin beta chain, non-erythrocytic 4 | SPTN4_HUMAN | 5.72 | 290005 | 15 | 63 |
| 9 | 1487 | P01834 | Immunoglobulin kappa constant | IGKC_HUMAN | 5.58 | 11773 | 56 | 61 |
| 10 | 1470 | P02647 | Apolipoprotein A-I | APOA1_HUMAN | 5.56 | 30759 | 72 | 160 |
| 11 | 382 | P02787 | Serotransferrin | TRFE_HUMAN | 6.82 | 79280 | 27 | 97 |
| 12 | 1136 | O75636 | Ficolin-3 | FCN3_HUMAN | 6.20 | 33395 | 39 | 80 |
| 13 | 7 | P02774 | Vitamin D-binding protein | VTDB_HUMAN | 5.40 | 54526 | 49 | 151 |
| 14 | 865 | P00738 | Haptoglobin | HPT_HUMAN | 6.13 | 45861 | 27 | 91 |
| 15 | 864 | P01024 | Complement C3 | CO3_HUMAN | 6.02 | 188569 | 16 | 60 |
| 16 | 1322 | P00739 | Haptoglobin-related protein | HPTR_HUMAN | 6.63 | 39518 | 25 | 63 |
| 17 | 857 | Q07065 | Cytoskeleton-associated protein 4 | CKAP4_HUMAN | 5.63 | 66097 | 18 | 57 |
| 18 | 385 | P02787 | Serotransferrin | TRFE_HUMAN | 6.82 | 79280 | 62 | 234 |
| 19 | 820 | Q14585 | Zinc finger protein 345 | ZN345_HUMAN | 8.70 | 57229 | 26 | 56 |
| 20 | 1427 | Q9BZ29 | Dedicator of cytokinesis protein 9 | DOCK9_HUMAN | 7.25 | 238519 | 16 | 65 |
| 21 | 1120 | O75648 | Mitochondrial tRNA-specific 2-thiouridylase 1 | MTU1_HUMAN | 8.24 | 48284 | 21 | 57 |
| 22 | 1759 | P02766 | Transthyretin | TTHY_HUMAN | 5.52 | 15991 | 69 | 135 |
| 23 | 1193 | P04264 | Keratin, type II cytoskeletal 1 | K2C1_HUMAN | 8.15 | 66170 | 34 | 85 |
| 24 | 108 | O75121 | Microfibrillar-associated protein 3-like | MFA3L_HUMAN | 5.13 | 45750 | 19 | 58 |

| | | | | | | | | |
|---|---|---|---|---|---|---|---|---|
| 25 | 185 | P01011 | Alpha-1-antichymotrypsin | AACT_HUMAN | 5.33 | 47792 | 24 | 64 |
| 26 | 266 | P02768 | Albumin | ALBU_HUMAN | 5.92 | 71371 | 53 | 133 |
| 27 | 235 | P17039 | Zinc finger protein 30 | ZNF30_HUMAN | 9.33 | 64035 | 34 | 62 |
| 28 | 357 | P08631 | Tyrosine-protein kinase HCK | HCK_HUMAN | 6.27 | 60075 | 22 | 56 |
| 29 | 661 | P01009 | Alpha-1-antitrypsin | A1AT_HUMAN | 5.37 | 46878 | 17 | 58 |
| 30 | 334 | P02790 | Hemopexin | HEMO_HUMAN | 6.55 | 52385 | 36 | 131 |
| 31 | 450 | P01009 | Alpha-1-antitrypsin | A1AT_HUMAN | 5.37 | 46878 | 49 | 133 |
| 32 | 1563 | P18206 | Vinculin | VINC_HUMAN | 5.50 | 124292 | 11 | 666 |
| 33 | 853 | P00738 | Haptoglobin | HPT_HUMAN | 6.13 | 45861 | 36 | 132 |
| 34 | 1448 | Q6ZNG0 | Zinc finger protein 620 | ZN620_HUMAN | 8.64 | 49612 | 22 | 61 |
| 35 | 1473 | Q9HBT8 | Zinc finger protein 286A | Z286A_HUMAN | 8.64 | 61505 | 22 | 58 |
| 36 | 1231 | P13645 | Keratin, type I cytoskeletal 10 | K1C10_HUMAN | 5.13 | 59020 | 28 | 82 |
| 37 | 548 | P02765 | Alpha-2-HS-glycoprotein | FETUA_HUMAN | 5.43 | 40098 | 33 | 60 |
| 38 | 497 | P01009 | Alpha-1-antitrypsin | A1AT_HUMAN | 5.37 | 46878 | 40 | 88 |
| 39 | 1784 | P02753 | Retinol-binding protein 4 | RET4_HUMAN | 5.76 | 23337 | 76 | 74 |
| 40 | 1475 | Q3L8U1 | Chromodomain-helicase-DNA-binding protein 9 | CHD9_HUMAN | 6.56 | 327986 | 8 | 57 |
| 41 | 1295 | Q969Q6 | Serine/threonine-protein phosphatase 2A regulatory subunit B" subunit gamma | P2R3C_HUMAN | 5.07 | 53567 | 26 | 60 |
| 42 | 1260 | P02647 | Apolipoprotein A-I | APOA1_HUMAN | 5.56 | 30759 | 73 | 225 |
| 43 | 636 | Q99661 | Kinesin-like protein KIF2C | KIF2C_HUMAN | 8.03 | 82174 | 18 | 57 |
| 44 | 1962 | P13645 | Keratin, type I cytoskeletal 10 | K1C10_HUMAN | 5.13 | 59020 | 17 | 63 |
| 45 | 456 | Q9HAC8 | Ubiquitin domain-containing protein 1 | UBTD1_HUMAN | 7.68 | 26150 | 16 | 57 |
| 46 | 1705 | O15226 | NF-kappa-B-repressing factor | NKRF_HUMAN | 8.94 | 78308 | 16 | 60 |
| 47 | 1132 | Q92896 | Golgi apparatus protein 1 | GSLG1_HUMAN | 6.52 | 138341 | 31 | 61 |
| 48 | 1877 | P20472 | Parvalbumin alpha | PRVA_HUMAN | 4.98 | 12051 | 58 | 68 |
| 49 | 504 | Q08999 | Retinoblastoma-like protein 2 | RBL2_HUMAN | 7.27 | 129711 | 26 | 73 |
| 50 | 1484 | P02768 | Albumin | ALBU_HUMAN | 5.92 | 71371 | 80 | 230 |
| 51 | 1277 | O75636 | Ficolin-3 | FCN3_HUMAN | 6.20 | 33395 | 44 | 64 |
| 52 | 354 | O60384 | Putative zinc finger protein 861 | YS022_HUMAN | 8.91 | 12388 | 59 | 59 |
| 53 | 650 | P01009 | Alpha-1-antitrypsin | A1AT_HUMAN | 5.37 | 46878 | 40 | 106 |
| 54 | 1037 | Q14683 | Structural maintenance of chromosomes protein 1A | SMC1A_HUMAN | 7.51 | 143771 | 26 | 59 |
| 55 | 1138 | Q6ZN19 | Zinc finger protein 841 | ZN841_HUMAN | 9.48 | 95939 | 28 | 62 |
| 56 | 1669 | P02753 | Retinol-binding protein 4 | RET4_HUMAN | 5.76 | 23337 | 43 | 70 |
| 57 | 1446 | Q9BV73 | Centrosome-associated protein CEP250 | CP250_HUMAN | 5.00 | 281880 | 19 | 58 |
| 58 | 886 | P00738 | Haptoglobin | HPT_HUMAN | 6.13 | 45861 | 19 | 58 |

| | | | | | | | | |
|---|---|---|---|---|---|---|---|---|
| 59 | 457 | Q96M63 | Coiled-coil domain-containing protein 114 | CC114_HUMAN | 5.87 | 75172 | 24 | 67 |
| 60 | 522 | Q9Y473 | Zinc finger protein 175 | ZN175_HUMAN | 8.98 | 84009 | 41 | 82 |
| 61 | 1305 | Q12852 | Mitogen-activated protein kinase kinase kinase 12 | M3K12_HUMAN | 6.03 | 94130 | 11 | 56 |
| 62 | 1394 | Q14204 | Cytoplasmic dynein 1 heavy chain 1 | DYHC1_HUMAN | 6.01 | 534804 | 16 | 68 |
| 63 | 48 | P49792 | E3 SUMO-protein ligase RanBP2 | RBP2_HUMAN | 5.85 | 362365 | 14 | 65 |
| 64 | 903 | O95389 | Cellular communication network factor 6 | WISP3_HUMAN | 8.87 | 41319 | 28 | 66 |
| 65 | 28 | Q14624 | Inter-alpha-trypsin inhibitor heavy chain H4 | ITIH4_HUMAN | 6.51 | 103521 | 26 | 81 |
| 66 | 1083 | Q9UKX3 | Myosin-13 | MYH13_HUMAN | 5.56 | 224681 | 19 | 65 |
| 67 | 1067 | Q9NQW7 | Xaa-Pro aminopeptidase 1 | XPP1_HUMAN | 5.42 | 70557 | 26 | 57 |
| 68 | 423 | Q5R372 | Rab GTPase-activating protein 1-like | RBG1L_HUMAN | 5.18 | 70557 | 26 | 57 |
| 69 | 1213 | Q14683 | Structural maintenance of chromosomes protein 1A | SMC1A_HUMAN | 7.51 | 143771 | 29 | 63 |
| 70 | 1180 | Q8TED0 | U3 small nucleolar RNA-associated protein 15 homolog | UTP15_HUMAN | 9.18 | 58661 | 30 | 70 |
| 71 | 805 | P04637 | Cellular tumor antigen p53 | P53_HUMAN | 6.33 | 44196 | 22 | 56 |
| 72 | 388 | P00751 | Complement factor B | CFAB_HUMAN | 6.67 | 86847 | 36 | 95 |
| 73 | 1106 | Q8TD57 | Dynein heavy chain 3, axonemal | DYH3_HUMAN | 6.04 | 473776 | 14 | 64 |
| 74 | 579 | P02768 | Albumin | ALBU_HUMAN | 5.92 | 71371 | 48 | 104 |
| 75 | 215 | Q9Y4I1 | Unconventional myosin-Va | MYO5A_HUMAN | 8.70 | 216995 | 22 | 64 |
| 76 | 1865 | P02766 | Transthyretin | TTHY_HUMAN | 5.52 | 15991 | 69 | 58 |
| 77 | 953 | P00738 | Haptoglobin | HPT_HUMAN | 6.13 | 45861 | 39 | 92 |
| 78 | 528 | P02768 | Albumin | ALBU_HUMAN | 5.92 | 71371 | 45 | 68 |
| 79 | 1627 | P02647 | Apolipoprotein A-I | APOA1_HUMAN | 5.56 | 30759 | 33 | 76 |
| 80 | 1659 | P02647 | Apolipoprotein A-I | APOA1_HUMAN | 5.56 | 30759 | 69 | 157 |
| 81 | 993 | Q7Z398 | Zinc finger protein 550 | ZN550_HUMAN | 8.95 | 49491 | 25 | 61 |
| 82 | 562 | P19652 | Alpha-1-acid glycoprotein 2 | A1AG2_HUMAN | 5.03 | 23873 | 42 | 85 |
| 83 | 974 | P35558 | Phosphoenolpyruvate carboxykinase, cytosolic [GTP] | PCKGC_HUMAN | 5.80 | 69948 | 23 | 66 |
| 84 | 990 | Q5BJF6 | Outer dense fiber protein 2 | ODFP2_HUMAN | 7.53 | 96140 | 30 | 59 |
| 85 | 1211 | P04264 | Keratin, type II cytoskeletal 1 | K2C1_HUMAN | 8.15 | 66170 | 36 | 90 |
| 86 | 1054 | Q9H792 | Inactive tyrosine-protein kinase PEAK1 | SG269_HUMAN | 6.46 | 195039 | 18 | 71 |
| 87 | 955 | Q5BJF6 | Outer dense fiber protein 2 | ODFP2_HUMAN | 7.53 | 96140 | 36 | 93 |

| | | | | | | | | |
|---|---|---|---|---|---|---|---|---|
| 88 | 2217 | P68871 | Hemoglobin subunit beta | HBB_HUMAN | 6.76 | 16102 | 63 | 57 |
| 89 | 1039 | P78332 | RNA-binding protein 6 | RBM6_HUMAN | 5.93 | 129192 | 26 | 70 |
| 90 | 913 | P02538 | Keratin, type II cytoskeletal 6A | K2C6A_HUMAN | 8.09 | 60239 | 35 | 80 |
| 91 | 604 | Q6AW86 | Zinc finger protein 324B | Z324B_HUMAN | 9.78 | 61818 | 34 | 60 |
| 92 | 981 | P06727 | Apolipoprotein A-IV | APOA4_HUMAN | 5.28 | 45371 | 41 | 128 |
| 93 | 1908 | P02647 | Apolipoprotein A-I | APOA1_HUMAN | 5.56 | 30759 | 49 | 100 |
| 94 | 2323 | P02042 | Hemoglobin subunit delta | HBD_HUMAN | 7.85 | 16159 | 63 | 61 |
| 95 | 481 | Q5R372 | Rab GTPase-activating protein 1-like | RBG1L_HUMAN | 5.18 | 93366 | 16 | 59 |
| 96 | 623 | Q92608 | Dedicator of cytokinesis protein 2 | DOCK2_HUMAN | 6.43 | 213123 | 16 | 59 |
| 97 | 771 | Q96EH8 | E3 ubiquitin-protein ligase NEURL3 | LINCR_HUMAN | 8.36 | 29626 | 38 | 65 |
| 98 | 341 | Q9P219 | Protein Daple | DAPLE_HUMAN | 5.87 | 229215 | 15 | 70 |
| 99 | 796 | P00738 | Haptoglobin | HPT_HUMAN | 6.13 | 45861 | 33 | 95 |
| 100 | 987 | Q709C8 | Vacuolar protein sorting-associated protein 13C | VP13C_HUMAN | 6.38 | 424462 | 12 | 68 |
| 101 | 732 | P25311 | Zinc-alpha-2-glycoprotein | ZA2G_HUMAN | 5.57 | 34079 | 37 | 57 |
| 102 | 702 | Q9UM13 | Anaphase-promoting complex subunit 10 | APC10_HUMAN | 9.17 | 21410 | 22 | 63 |
| 103 | 533 | P02774 | Vitamin D-binding protein | VTDB_HUMAN | 5.40 | 54526 | 26 | 69 |
| 104 | 1465 | P17017 | Zinc finger protein 14 | ZNF14_HUMAN | 9.36 | 77970 | 37 | 67 |
| 105 | 788 | A8TX70 | Collagen alpha-5(VI) chain | CO6A5_HUMAN | 6.50 | 291796 | 15 | 58 |
| 106 | 584 | Q8TD57 | Dynein heavy chain 3, axonemal | DYH3_HUMAN | 6.04 | 473776 | 13 | 60 |
| 107 | 1688 | P20472 | Parvalbumin alpha | PRVA_HUMAN | 4.98 | 12051 | 57 | 56 |
| 108 | 633 | P02768 | Albumin | ALBU_HUMAN | 5.92 | 71371 | 62 | 176 |
| 109 | 709 | Q9NZM1 | Myoferlin | MYOF_HUMAN | 5.84 | 236100 | 18 | 70 |
| 110 | 198 | P02768 | Albumin | ALBU_HUMAN | 5.92 | 71371 | 59 | 245 |
| 111 | 743 | P01009 | Alpha-1-antitrypsin | A1AT_HUMAN | 5.37 | 46878 | 40 | 102 |
| 112 | 676 | P02749 | Beta-2-glycoprotein 1 | APOH_HUMAN | 8.34 | 39584 | 51 | 58 |
| 113 | 556 | P20929 | Nebulin | NEBU_HUMAN | 9.11 | 775419 | 14 | 64 |
| 114 | 537 | P02768 | Albumin | ALBU_HUMAN | 5.92 | 71371 | 53 | 114 |
| 115 | 714 | Q02156 | Protein kinase C epsilon type | KPCE_HUMAN | 6.73 | 84989 | 24 | 71 |
| 116 | 588 | Q96SZ6 | Mitochondrial tRNA methylthiotransferase CDK5RAP1 | CK5P1_HUMAN | 8.52 | 68501 | 12 | 57 |
| 117 | 275 | Q9Y473 | Zinc finger protein 175 | ZN175_HUMAN | 8.98 | 84009 | 22 | 58 |
| 118 | 797 | P00738 | Haptoglobin | HPT_HUMAN | 6.13 | 45861 | 35 | 94 |
| 119 | 768 | O76041 | Nebulette | NEBL_HUMAN | 7.89 | 116609 | 21 | 62 |
| 120 | 550 | Q8TE73 | Dynein heavy chain 5, axonemal | DYH5_HUMAN | 5.97 | 532504 | 17 | 60 |
| 121 | 549 | P51508 | Zinc finger protein 81 | ZNF81_HUMAN | 8.96 | 77679 | 29 | 67 |

[a] Spot number.
[b] Protein accession number for SWISSPROT Database.
[c] Theoretical isoelectric point.
[d] Theoretical relative mass.
[e] MASCOT score

**Table S3:** List of 25 proteins with accession numbers depicted in IPA network pathway

| Sl no | Accession No | Protein Name | MASCOT ID |
|---|---|---|---|
| 1 | Q9Y4I1 | Unconventional myosin-Va | MYO5A_HUMAN |
| 2 | P13645 | Keratin, type I cytoskeletal 10 (KRT10*) | K1C10_HUMAN |
| 3 | Q08999 | Retinoblastoma-like protein 2 | RBL2_HUMAN |
| 4 | P04637 | Cellular tumor antigen p53 (**TP53)** | P53_HUMAN |
| 5 | Q9Y473 | Zinc finger protein 175 | ZN175_HUMAN |
| 6 | P01011 | Alpha-1-Antichymotrypsin (SERPINA3) | AACT_HUMAN |
| 7 | P18206 | Vinculin (VCL) | VINC_HUMAN |
| 8 | P08631 | Tyrosine-protein kinase (HCK) | HCK_HUMAN |
| 9 | Q02156 | Protein kinase C epsilon Type (PRKCE) | KPCE_HUMAN |
| 10 | O15226 | NF-kappa-B-repressing factor | NKRF_HUMAN |
| 11 | P21580 | Tumor necrosis factor alpha-induced protein 3 (TNFAIP3) | TNAP3_HUMAN |
| 12 | P02753 | Retinol-binding protein 4 (RBP4) | RET4_HUMAN |
| 13 | P25311 | Zinc-alpha-2-Glycoprotein (AZGP1) | ZA2G_HUMAN |
| 14 | P02766 | Transthyretin (TTR*) | TTHY_HUMAN |
| 15 | P01024 | Complement C3 (C3) | CO3_HUMAN |
| 16 | P00751 | Complement factor B (CFB) | CFAB_HUMAN |
| 17 | P02787 | Serotransferrin (TF) | TRFE_HUMAN |
| 18 | P02768 | Albumin (ALB) | ALBU_HUMAN |
| 19 | P68871 | Hemoglobin subunit beta | HBB_HUMAN |
| 20 | P02647 | Apolipoprotein A-I | APOA1_HUMAN |
| 21 | Q8TD57 | Dynein heavy chain 3, Axonemal (DYNC1H1) | DYH3_HUMAN |

| | | | |
|---|---|---|---|
| 22 | P02790 | Hemopexin (HPX) | HEMO_HUMAN |
| 23 | P06727 | Apolipoprotein A-IV | APOA4_HUMAN |
| 24 | P00738 | Haptoglobin (HP) | HPT_HUMAN |
| 25 | O95389 | Cellular communication network factor 6 (CCN6) | WISP3_HUMAN |

**Table S4:** List of proteins with accession numbers for the top 5 canonical pathways

| | CANONICAL PATHWAYS | ACCESSION NUMBER | PROTEIN NAME |
|---|---|---|---|
| 1 | **LXR/RXR Activation** | P02765 | Alpha-2-HS-glycoprotein |
| | | P02768 | Albumin |
| | | P02647 | Apolipoprotein A-I |
| | | P06727 | Apolipoprotein A-IV |
| | | P02749 | Beta-2-glycoprotein 1 |
| | | P01024 | Complement C3 |
| | | P02774 | Vitamin D-binding protein |
| | | P00739 | Haptoglobin-related protein |
| | | P02790 | Hemopexin |
| | | Q14624 | Inter-alpha-trypsin inhibitor heavy chain H4 |
| | | P19652 | Alpha-1-acid glycoprotein 2 |
| | | P02753 | Retinol-binding protein 4 |
| | | P01009 | Alpha-1-antitrypsin |
| | | P02787 | Serotransferrin |
| | | P02766 | Transthyretin |
| 2 | **FXR/RXR Activation** | P02765 | Alpha-2-HS-glycoprotein |
| | | P02768 | Albumin |
| | | P02647 | Apolipoprotein A-I |
| | | P06727 | Apolipoprotein A-IV |
| | | P02749 | Beta-2-glycoprotein 1 |
| | | P01024 | Complement C3 |
| | | P02774 | Vitamin D-binding protein |
| | | P00739 | Haptoglobin-related protein |
| | | P02790 | Hemopexin |
| | | Q14624 | Inter-alpha-trypsin inhibitor heavy chain H4 |
| | | P19652 | Alpha-1-acid glycoprotein 2 |
| | | P02753 | Retinol-binding protein 4 |
| | | P01009 | Alpha-1-antitrypsin |
| | | P02787 | Serotransferrin |
| | | P02766 | Transthyretin |
| 3 | **Acute Phase Response Signaling** | P02765 | Alpha-2-HS-glycoprotein |
| | | P02768 | Albumin |
| | | P02647 | Apolipoprotein A-I |

| | | | |
|---|---|---|---|
| | | P02749 | Beta-2-glycoprotein 1 |
| | | P01024 | Complement C3 |
| | | P00751 | Complement factor B |
| | | P00738 | Haptoglobin |
| | | P02790 | Hemopexin |
| | | Q14624 | Inter-alpha-trypsin inhibitor heavy chain H4 |
| | | P19652 | Alpha-1-acid glycoprotein 2 |
| | | P02753 | Retinol-binding protein 4 |
| | | P01009 | Alpha-1-antitrypsin |
| | | P01011 | Alpha-1- antichymotrypsin |
| | | P02787 | Serotransferrin |
| | | P02766 | Transthyretin |
| 4 | **Production of Nitric oxide and Reactive Oxygen Species** | P02768 | Albumin |
| | | P02647 | Apolipoprotein A-I |
| | | P06727 | Apolipoprotein A-IV |
| | | Q12852 | Mitogen-activated protein kinase kinase kinase 12 |
| | | P19652 | Alpha-1-acid glycoprotein 2 |
| | | Q02156 | Protein kinase C epsilon type |
| | | P02753 | Retinol-binding protein 4 |
| | | P01009 | Alpha-1-antitrypsin |
| 5 | **Atherosclerosis Signaling** | P02768 | Albumin |
| | | P02647 | Apolipoprotein A-I |
| | | P06727 | Apolipoprotein A-IV |
| | | Q92896 | Golgi apparatus protein 1 |
| | | P19652 | Alpha-1-acid glycoprotein 2 |
| | | P02753 | Retinol-binding protein 4 |
| | | P01009 | Alpha-1-antitrypsin |
